# The Role of Anions in Rare-earth Activated Inorganic Host Materials for its Luminescence Characteristics

**DOI:** 10.1007/s10895-023-03561-0

**Published:** 2024-02-13

**Authors:** Leelakrishna Reddy

**Affiliations:** Department of Physics, University of Johannesburg, Johannesburg, 2006 South Africa

**Keywords:** Phosphor, Luminescence, WLEDs, Dopants, Energy transfer

## Abstract

This work is inspired from the comprehensive work done by our research team aimed at improving the efficiency of white light emitting diodes (LEDs) through improvements in the colour rendering index of the red light (CRI), one of the primary colours of white light. Such work is triggered through the incorporation of anions (BO_3_^3−^, PO_4_^3−^, SO_4_^2−^), either individually or as an integral part of dopant activated inorganic phosphor host materials. Numerous host materials such as ZnO, Y_2_O_3_, Ca_3_(PO_4_)_2_, CaMoO_4_, ABPO_4_, ABSO_4_ (where A represents alkali metals and B alkaline earth metals) have been considered ideal hosts materials for studying luminescence properties of materials (including other phosphors). In addition, red emitting dopants such as Sm^3+^, Eu^3+^ and Ce^3+^ have been incorporated into these host materials to achieve a higher CRI of red colour, an essential component of white light. The role anions in various materials is multifaceted; firstly, it acts as sensitizer whereby it absorbs excitation energy and transfers it non-radiatively to the dopants, secondly, it acts as a charge compensator to dopants with a charge of + 3, thirdly, it creates crystal fields that affects the electronic transitions of the dopants and fourthly, it creates a stable crystal structure that allows for dopant embedding. By understanding the exact role of these anions and their interactions with the host lattice and dopant ions, we could further optimize the luminescent properties of these activated host materials, which leads to higher efficiencies and performances in white light-emitting diodes and other lighting technologies. This work is a comprehensive review of the work undertaken by our research team aimed at enhancing the luminescent properties of WLEDs.

## Introduction

The pursuit of exceptionally efficient white light-emitting diodes (LEDs) has driven researchers worldwide towards this strategic goal. This review is aimed at augmenting one of the fundamental constituents of white light, namely, red light, which presents a challenge in achieving a superior color rendering index (CRI) [1, 2]. In pursuit of such research endeavours, anions such as BO_3_^3−^, PO_4_^3−^, and SO_4_^2−^ have emerged as potential agencies for modifying the luminescent characteristics of inorganic phosphor host materials, either substituted individually or infused as part of the host lattice matrix. The inspiration of this review work stems from the comprehensive research conducted by our group over several years (including other research work as well), that was is aimed at fabricating materials that could surpass the efficiency of the commercially available WLEDs, by adjusting mainly the cationic metallic ions (Ca^2+^, Ba^2+^, Sr^2+^ and Mg^2+^), and with anions as potential modifiers.

Anions (BO_3_^3−^, PO_4_^3−^, and SO_4_^2−^) that have been incorporated into the phosphor host material, either through ionic interactions or as an integral component, have been found to remarkably enhance its photoluminescence intensities, resulting from its unique crystal structure formation [3]. Such observations were noted when PO_4_^3−^ was incorporated into Eu^3+^ -activated Y_2_O_3_ hosts, producing a 5 -fold increase in the luminescent intensity than without the anionic presence [4]. In another instance, when other SO_4_^2−^ or BO_3_^3−^ anions were incorporated into the dopant activated hosts, high photoluminescent intensities were also observed [5, 6]. In the research by Balakrishna et al. [3], it was observed that when the anions were individually incorporated into the host material, the luminescent intensity of CaMoO_4_-SO_4_ improved 30-fold, followed by CaMoO_4_-BO_3_ and CaMoO_4_-PO_4_ materials with relatively high intensities in that order, confirming the enhancing roles played by all these anions. Furthermore, anionic incorporation was found to decrease the band gap of the host material and improve its quantum yield efficiency. Each of these anions play a unique role when individually incorporated into different host materials. For instance, the additions of BO_3_^3−^ was found to play a dual role in host materials, acting either as a sensitizer or an activator. When acting as a sensitizer, it absorbs energy from the excitation source and transfers it non-radiatively to the activator ions, which then re-emits it as light [1]. It can create crystal fields that influence the electronic transitions of the dopants. Anions typically act as charge compensator in various host materials. On the other hand, the phosphate group (PO_4_^3−^) with its tetrahedral crystal structure, influences the luminescence in different ways: it can facilitate the energy transfer to dopants, thereby acting as a sensitizer, and it can further create crystal fields that influences the electronic transitions of the dopant ions [7]. Other anions, such as SO_4_^2−^, which share a similar tetrahedral structure with the phosphate group PO_4_^3−^, essentially perform the same functions as PO_4_^3−^. Phosphates also serve as charge compensators, creating crystal fields that influence the electronic transitions of dopants and position itself within the host material in such a way to minimize luminescence quenching effects [8].

In this study, various host phosphor materials, such as ZnO, Y_2_O_3_, Ca_3_(PO_4_)_2_, CaMoO_4_, CaLi(PO_4_)_2-x_(SO_4_)_x_, LiY(PO_3_)_4_, ABPO_4_, ABSO_4_ (where A represents alkali metal ions and B represents alkaline earth metal ions), have been explored for the incorporation of various anionic group systems. They have been primarily considered for their individual substitutions or integral infusion into inorganic phosphor host material.

In this review work, dopants such as Eu^3+^ have been used for their strong red emitting properties when incorporated into various host materials. Other dopants, such as Sm^3+^ have been employed as a red to orange-red emitting phosphor material when embedded in different host materials. On the other hand, Ce^3+^ which typically emits violet-blue emissions can be tuned to shift its emission to the red end of the spectrum by concentration variation. The primary red colour, combined with primary and/or secondary colours produce white light required in WLEDS. Furthermore, the interactions of these anions (BO_3_^3−^, PO_4_^3−^, and SO_4_^2−^) with the host material can tune its emission characteristics, which are remarkably high compared to without their absence. As a result, these anions act as a catalyst in optimizing the luminescence characteristics of these dopant-activated phosphor host materials. Their strategic presence in dopant-activated material is crucial for their luminescent characteristics. In this paper, we will explore the significant role played by each of these anions (BO_3_^3−^, PO_4_^3−^ and SO_4_^2−^) in various host materials and their impact on emission properties. By systematically reviewing the research done on different host materials, we will then be able to fully comprehend the role they have played in enhancing the emission properties in dopant- activated host materials. Such findings will pave the way for the development of super-efficient phosphor materials for the next generation of WLED applications.

### Enhancing White LED Performance: Resolving the Interplay of LED Theory and Anionic Substitutions in Semiconductor Phosphor Material

Incandescent light bulbs, such as those developed by Thomas Edison, convert electrical energy indirectly to heat energy before producing light. On the other hand, fluorescent lamps essentially work by allowing an electric current to pass through an encapsulated gas, producing ultraviolet emissions that interact with a phosphorescent coating on the inner surface to generate visible light. Unlike traditional incandescent and fluorescent lighting, Light Emitting Diodes (LEDs) directly convert electrical energy to light energy, making them more energy efficient. The architecture of an LED system consists of three layers superimposed upon each other: a p-type (positive) semiconductor, an n-type (negative) semiconductor, and an active or depletion layer sandwiched between these two layers. When a potential difference is applied, and the LED is in forward bias mode, free electrons (from the n-type semiconductor) and holes (from the p-type semiconductor) merge at the depletion layer. Their recombination results in the emission of photons or light. In terms of band theory, free electrons from the semiconductor conduction band recombine with holes from the semiconductor valence band, leading to the emission of photons or monochromatic light [6].

In essence, there are two types of solid-state LED lighting systems: semiconducting inorganic LEDs and semiconducting organic LEDs (OLEDs). The design architecture of these systems differs significantly. Previous discussions have primarily focused on LED composition and operation. In addition to this, the doping of semiconducting n-regions introduces negative electrons, and their combination with positive charges (holes) results in light emission under forward voltage bias. On the other hand, in OLEDs, the structure consists of an anode (positive) and a cathode (negative) sandwiched by an organic material and mounted on a substrate at the cathode side. The emission of light in OLEDs occurs due to a hopping process by positive holes along the electric field lines across the highest occupied molecular levels and the opposite movement of electrons across the lowest unoccupied levels of the material. Their meeting point in the organic material leads to the emission of light when they return to a lower energy state after excitation [6].

LEDs have many advantages such as [6–8]: (a) their brightness can be adjusted through current variations, thus enabling colour tuning, (b) they consume low power, thus making them to be energy efficient lighting sources, (c) they are cost effective and easily available in the market, (d) they are compact and lightweight owing to their small sizes, (e) they have longer lifetimes compared to conventional light sources, (f) they are devoid of toxic materials and thus are environmentally friendly, (g) they emit a variety of colours dependent on the dopant material used, (h) they are used mostly in street lighting, floodlights, indoor lighting, colour displays systems, traffic lights, exit lights in cinemas and television monitors. Besides these advantages, they do have some disadvantages; namely [6, 9], (a) they have a low luminous efficiency compared to other lighting sources, (b) they require more power than normal p–n junction diodes, (c) their inability to provide steady illumination from DC/AC sources, (d) they have limitations in operating at lesser maximum temperatures and at storage temperatures, and (e) since the LED current is kept constant, an increase in temperature will cause a decrease in the LED performance.

In contrast, OLEDS have other advantages such as [6, 10]: (a) they are thin and flexible, thereby allowing unique design fabrication (b) they have a fast response time, (c) their luminescent flux is independent of temperature variations (large surface area), (d) they are operational at lower power densities compared to other lighting systems, (e) they display excellent colour accuracy, thereby producing high quality image reproduction, (f) they are commonly used for decorative and mood-lighting applications and (g) they are applied mostly in residential and street lighting due to their versatile nature. On the other hand, these OLEDs have some disadvantages such as: (a) they have a short life- time operation due to its organic structural make up, (b) their organic material is susceptible to exposure to oxygen and water, thereby are degraded over time, (c) they are generally more expensive to fabricate compared to inorganic LEDs.

Despite the advantages mentioned above, OLEDs still need some technological (such as electrical, thermal, architecture, durability, lifespan, etc.) advancement to make them more competitive in the industry [10]. The electrical behaviour of both inorganic LEDS and organic OLEDs are very similar because of the presence of the diode, but vary because of the existence of capacitors in OLEDs that makes them more complex to operate [10]. In terms of thermal behaviour, inorganic LEDs are very sensitive to temperature changes, whereas OLEDS are less sensitive to temperature variations [10]. Most companies today are investing in the creation of white light due to its versality and application in different colour settings [8]. After extensive research, it has become known that OLEDs are generally not suitable compared to LEDs when operating under harsh conditions [7]. On the other hand, inorganic white LEDs face many challenges in its design to achieve tunable CCT (correlated colour temperature) and excellent CRI values (colour rendering index) [11], implying that the emitted white light should replicate the sun’s high colour temperature during morning, midday, and afternoon times [11]. As such, it was determined from the research of [11] that this may be attained by having a warm white light, whose CCT value of 3183 K and compositions of red wavelength light of 634.1 nm, green wavelength light of 513.9 nm and blue wavelength light of 456.2 nm could correlate with a tunable CCT white light of CRI above 93 (greater than 80, as a benchmark of getting closer to natural sunlight) [11].

There are some technologies (not all) required to produce WLEDs [12]; namely, wavelength conversion and colour mixing. For wavelength conversion, the LED radiation is converted completely to white light. In this instance, there are many methods to produce white light; one of them is using a blue LED and a yellow phosphor material. In this method, the blue light emitted from the LED is used to excite a yellow (yttrium aluminum garnet, Y_3_Al_5_O_12_, doped with cerium) phosphor material, and the combination of which gives white light [7]. Another method is under wavelength conversion, is when a blue LED (radiation) is used in to excite several phosphor materials, resulting in different colour emissions, the combination of which with blue light creates white light colour emission. The last wavelength conversion occurs when UV emitted LED is used to excite red, blue, and green phosphors, and the combination of which produces white light. In addition to the above, one could also use blue light from an LED to excite quantum dots, thereby producing white light by their combinations. Colour mixing is another way to produce white light. In this process multiple LEDs (red, blue, and green) are encapsulated into a lamp and when combined in appropriate ratios produce white light. Since LEDs are used and not phosphor materials, there is no loss of energy, rendering them effective light sources [12].

It has become known that the most popular way of producing white LEDs is by combining a blue LED chip, consisting of InGaN, and a yellow emitting phosphor material Y_3_Al_5_O_12_:Ce^3+^, as mentioned above. This method of producing white light suffers from the lack of the red colour component, stemming from the white light produced having a low CRI (less than the benchmark value of 80) value and a high CCT value (greater than 7000 K) [13]. To ameliorate this situation, researchers then focused on exciting tricolour (RGB) phosphor materials with UV light to get white light [14]. Because of the interference between the different phosphor materials, this leads to uncoordinated emissions, poor light absorptions, low luminous efficiency, and higher fabrication costs [14]. Thus, large scale production of these LEDs would be counterproductive [14]. Other methods such as using different techniques to synthesis materials, rare-earth co-doping is another method, but the method we will be focusing on is *anionic substitutions* to enhance the luminescence efficiencies of phosphor materials [14]. Another way of enhancing the luminescent efficiencies is through structural substitutions [13]. As such, partial substitution using anionic group systems induces structural modifications, that alters the crystal fields and facilitates charge compensation of the material [14]. This, in turn, results in the enhancement of the luminescent properties exhibited by anionic induced phosphor materials that have been widely published [13]. Most researchers have used europium as a dopant (or others) for red colour emission in different host materials. Still research with red emitting phosphor materials is ongoing to achieve the appropriate red emitting phosphor material that has a high luminescent efficiency, high thermal and chemical stability, high CRI and high colour purity [13].

## Key Roles that Anions Play in Dopant Activated Inorganic Phosphor Host Materials

The role that negatively charged anions (BO_3_^3−^, PO_4_^3−^, SO_4_^2−^) play in inorganic host materials doped with rare earth ions are discussed as follows:

### Sensitization Effects

When anions acquire the role of being a sensitizer, they absorb energy from the excitation source and transfers it non-radiatively to emitting luminescent centers, thereby enhancing the visible emission colours produced.

### Luminescence Quenching Effects

These anions can also transfer the absorbed energy away to other anions or to defect sites within the vicinity of the luminescent centers, thereby leading to quenching of the emissions colours.

### Tuning of Emission Characteristics

If these anions are strategically positioned within the crystal lattice, they can effectively modify the electronic transitions of the dopant ions, leading to an array of emission colours.

### Crystal Field Effects

These negatively charged anions interact with the positively charged ions (dopants), resulting in the creation of crystal field effects surrounding the luminescent centers, which, in turn, affects the emission colors produced.

### Charge Stability Effects

These anions, by bringing about charge stability of the host material, allows for the formation stable crystal structures, which prevents the degradation of the emission colours produced over time.

## Characterization of Luminescent Phosphor Materials

The following techniques will be used to characterize the phosphor material, with anions playing a multi-faceted role:

### X-ray Powder Diffraction (XRD) Measurements

These measurements are conducted to ensure that the anions do not alter the diffraction patterns of the host compound, thereby ensuring crystal conformity. In cases where they do, emission from transformed crystal structures will be studied in context.

### Fourier Transform Infrared (FTIR) Measurements

FTIR measurements is regarded as a complementary tool to identifying signature absorption spectra of the anionic group structures.

### Photoluminescence Excitation (PLE) and Photoluminescence Emission (PL) Spectra

This very important characterization tool is used to study the electronic transitions of the dopant ions, where the anions play an enhancing role.

## Role of the Single Negative Phosphate Group (PO_4_^3−^) in the Sodium Orthophosphate Host NaMPO_4_:Eu^3+^ (M^2+^ = Mg, Ca, Sr, and Ba) Material [15]

The phosphate ion (PO_4_^3−^) group in this situation interacts with the positive Eu^3+^ ion, which creates crystal fields that modifies the electronic transitions of the dopant ions, thereby affecting the intensity of the red colours produced. The different ionic radius of the alkaline earth ions (cations) accounts for variations in the emissions properties of the phosphor host materials.

### Review of the Key Features of this Study

XRD results below reveal that NaMgPO_4_-Eu^3+^ and NaSrPO_4_-Eu^3+^phosphors produce monoclinic crystal structures, while NaCaPO_4_-Eu^3+^ and NaBaPO_4_-Eu^3+^phosphors produce orthorhombic crystal structures, when synthesized by the combustion method. There were minimal impurities produced when NaCaPO_4_ and NaBaPO_4_ samples were crystallized, but more impurities such as Na_3_PO_4_ and EuPO_4_ were formed in the diffraction patterns when NaMgPO_4_ and NaSrPO_4_ samples were crystallized. In the latter case the diffraction patterns shifted towards lower 2θ values. The addition of the dopant Eu^3+^ (0.94 Å) ion did not cause any significant change to the diffraction patterns due to the similarity of their ionic radius of Mg^2+^( 0.86 Å), Ca^2+^(1.12 Å), Sr^2+^(1.32 Å) and Ba^2+^(1.49 Å) when replaced through doping. The alkaline earth ions were responsible for transformation of crystal structures.

**Fig. 1:**
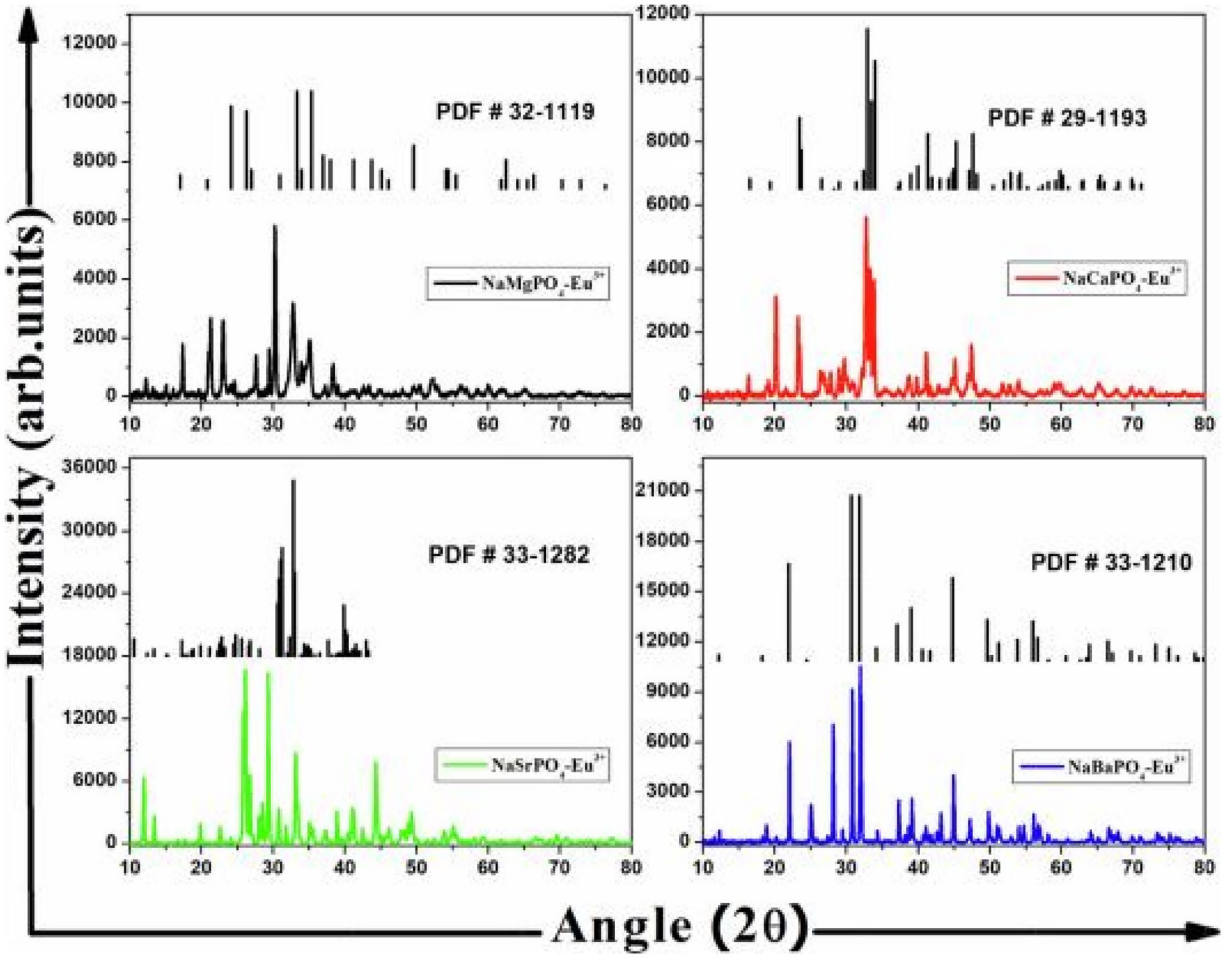
XRD patterns of the NaMPO_4-_Eu^3+^ phosphor materials with M^2+^ = Ca, Ba, Sr, and Mg [15] (Reused with permission from [15])

The radius of the dopant ion is compared to each of the substituent ions, and so deviation at the lattice site occurs when the dopant is either bigger or smaller than the substituent ion. This is obtained by using a formula [16] given as follows:

$${\Delta }_{r} = \left|\frac{{R}_{a}-{R}_{b}}{{R}_{a}}\right|x \,100\%\,,$$

and so, for a coordination number of CN = 6, the ionic radius of the following ions are given as follows: Na^+^ (1.02 Å), Ca^2+^ (1.00 Å), Mg^2+^(0.72 Å), Sr^2+^(1.18 Å), Ba^2+^(1.35 Å) and Eu^3+^(0.947 Å). This will give percentage deviations of 7.15%, 31.5%, 19.74%, 5.3% & 29.85% for Na^+^, Mg^2+^, Sr^2+^, Ca^2+^, and Ba^2+^ ions when respectively replaced by dopants. If CN = 8, then ${\Delta }_{r}$ will give values of 9.66%, 19.10%, 15.40%, 4.82% and 25.35% for Na^+^, Mg^2+^, Sr^2+^, Ca^2+^, and Ba^2+^ ions when respectively replaced by the dopant. These values, either for CN = 6 or 8, which are mostly less than 30% implies that Eu^3+^ could replace any one of them (Na^+^, Mg^2+^, Sr^2+^, Ca^2+^ and Ba^2+^ without causing too much crystal distortions [16], except for Mg^2+^ ion which has a percentage of slightly above 30%, when CN = 6 (see Fig. 1).

**Fig. 2:**
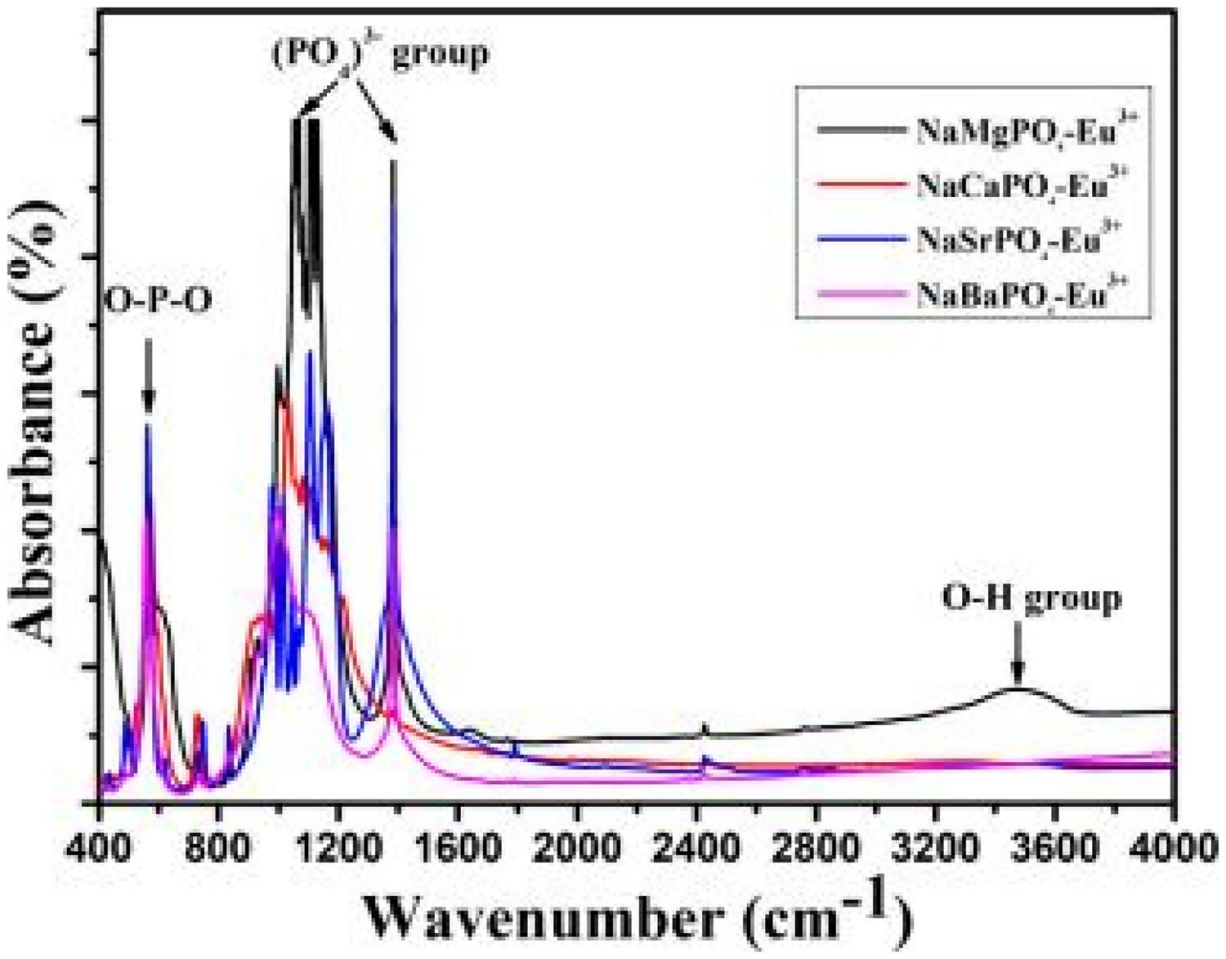
FTIR spectra of the NaMPO_4_-Eu^3+^ phosphor materials with M^2+^ = Ca, Ba, Sr, and Mg. The inset show the various series doped with Eu^3+^ ions [15] (Reused with permission from [15])

FTIR measurements below shows the absorptions properties of the phosphate group ions. We observe symmetric stretching vibrations of the PO_4_^3−^ phosphate groups, and symmetric bending vibrations of the O–P–O groups being prominent in this figure (see Fig. 2).

**Fig. 3:**
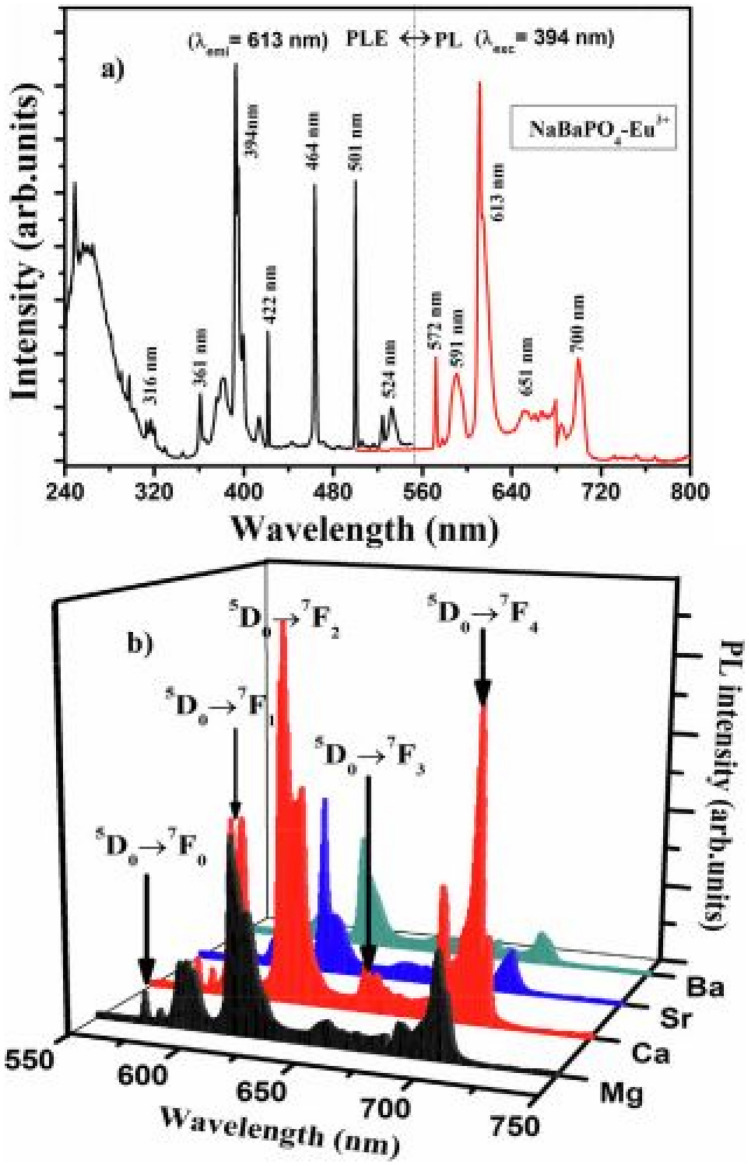
PLE and PL spectra of a sampler NaBaPO_4_:Eu^3+^ phosphor material is shown in figure (**a**) above, while the entire PL emission series for NaMPO_4_:Eu^3+^phosphors is shown in (**b**) [15] (Reused with permission from [15])

The PLE excitation and PL emission spectra of the NaBaPO_4_-Eu^3+^ phosphor material are shown below, as an example. The PLE excitation spectra shows a broad excitation spectrum between 240 and 320 nm, which is attributed to the charge transfer band (O^2−^ → Eu^3+^), as well as several f-f transition between 320 and 520 nm, which are due to transitions from the ground state of Eu^3+^ to higher excited states. In the PL emission spectra, we observe that the NaCaPO_4_-Eu^3+^ phosphor material produces the best red emissions, followed by NaMgPO_4_-Eu^3+^, NaSrPO_4_-Eu^3+^ and NaBaPO_4_-Eu^3+^ phosphor materials in that order. The cations appear to play a more significant role in luminescence while the inherent anions (PO_4_^3−^) appear to act as sensitizers (see Fig. 3).

### Important Finding of this Research

With the alkaline earth ion (cations) as tuning parameters in Eu^3+^ activated host materials, the strategic positioning of the phosphate group PO_4_^3−^ has the effect of further enhancing the red colour emissions produced through sensitizing and crystal field effects. Thus, NaCaPO_4_:Eu^3+^ phosphor material, with an orthorhombic crystal structure, produces the brightest red colour emission followed by Eu^3+^ doped NaMgPO_4_, NaSrPO_4_ and NaBaPO_4_ phosphor materials, respectively. The diverse crystal nature of the phosphate host materials (monoclinic to orthorhombic) further accounts for its unique emissions properties in transformed crystal structures. Eu^3+^ ions have been replaced by M^2+^ = Mg, Ba, Sr, Ca ions, as they act as tuning parameters in this research.

## Role of a Single Negative Sulphate Group (SO_4_^2−^) Substitution in the CaMoO_4_-Eu^3+^ Phosphor Material [17]

Sulphate anion (SO_4_^2−^) substitutions into Eu^3+^ activated calcium molybdate CaMoO_4_ host materials, modifies the crystal structure of the host by replacing the molybdate MoO^2−^ ions, and its presence with the dopants Eu^3+^ ions, creates crystal fields that alters the electronic transitions of the dopants for its red emitting properties. Such replacements were found to enhance the emission properties of the host lattice, producing high intensity red emissions, due to the dual presence of SO_4_^2−^ and Eu^3+^ ions. The replacement of Ca^2+^ by Eu^3+^ ions ensures charge stability of the host materials and significantly impacts on its luminescence features.

### Review of the Key Features of this Study

**Fig. 4:**
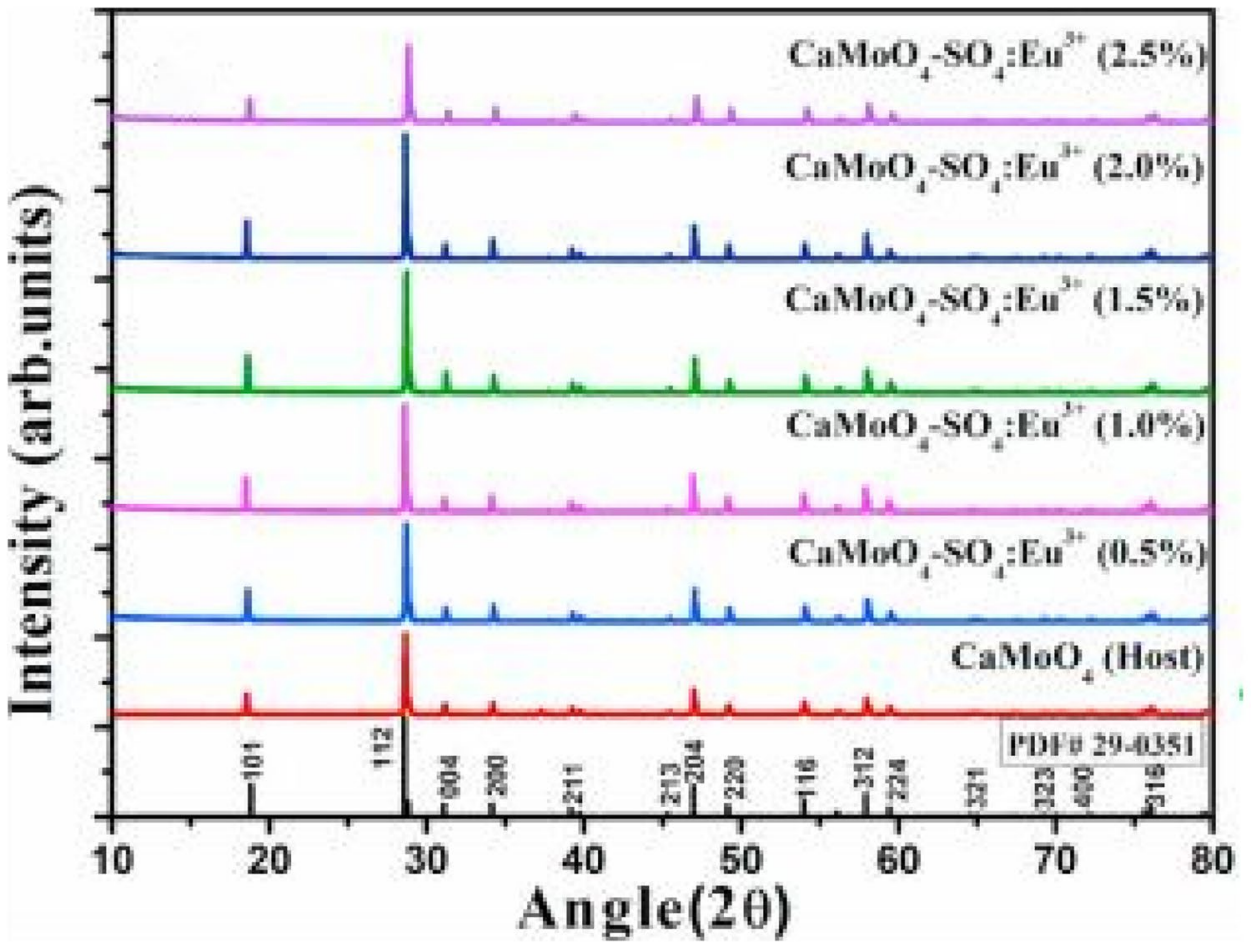
XRD patterns of the CaMoO_4_-SO_4_-xEu^3+^ (x = 0 to 2.5 mol%) phosphor materials [17] (Reused with permission from [17])

Figure 4 below shows the XRD diffraction patterns of the CaMoO_4_-SO_4_:xEu^3+^ phosphor materials for x = 0.5 to 2.5 mol%, prepared by the solid-state reaction method. All diffraction peaks were indexed to the standard scheelite tetragonal CaMoO_4_ crystal structure. The additions of the SO_4_^2−^ and Eu^3+^ ions has had a minimal effect on the diffraction patterns, except for slight changes either to the left or to right of 2θ when the concentrations of Eu^3+^ was varied, implying that good dissolution took place at the replacement sites of the Ca^2+^ and MoO_4_^2−^ ions by Eu^3+^ and SO_4_^2−^ ions, respectively.

The deviation percentage at lattice sites is obtained using the formula earlier in the section. For this section, ${\Delta }_{r}$ has a percentage of 5.3% when CN = 6 and a percentage of 4.82% when CN = 8. These values are less than 30%, implies that Eu^3+^ replaces Ca^2+^ with minimal distortions, but with imbalance of charges. On the other hand, the replacement of SO_4_^2−^ by MoO_4_^2−^ ions are largely possible since there appear to be no additional peaks in the XRD spectra. However, SO_4_^2−^ may interact with Eu^3+^ to produce crystal field effects, and/or cause shifts in intensity peak positions.

**Fig. 5:**
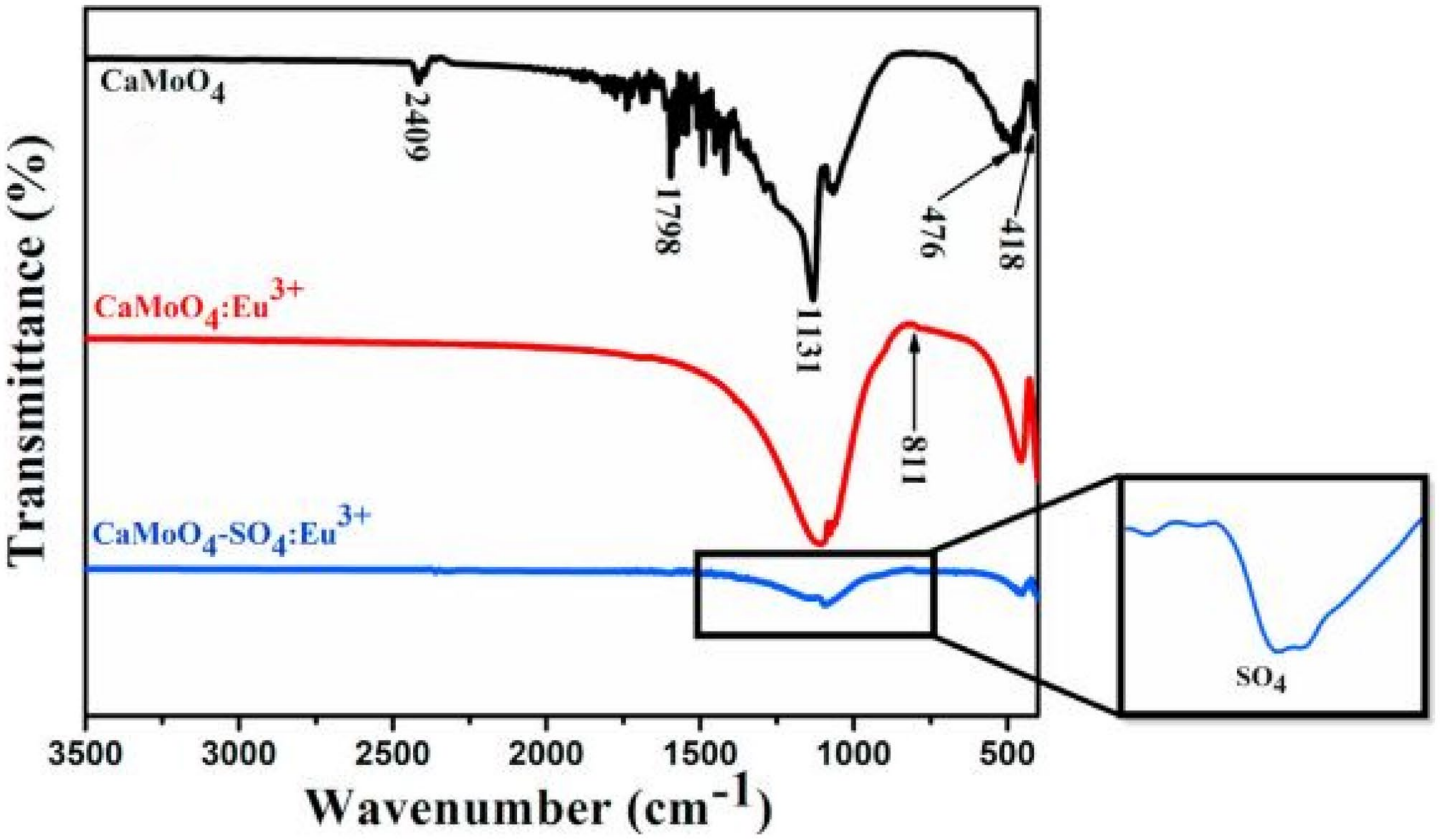
FTIR spectra of the CaMoO_4_-SO_4_-Eu^3+^ phosphor material [17] (Reused with permission from [17])

Figure 5 shows the FTIR measurements of the CaMoO_4_-SO_4_-Eu^3+^ phosphor material. The stretching modes of SO_4_^2−^ group and its absorption properties are shown in the figure. We observe a reduction in the absorption properties compared to the host material for SO_4_^2−^ additions.

**Fig. 6:**
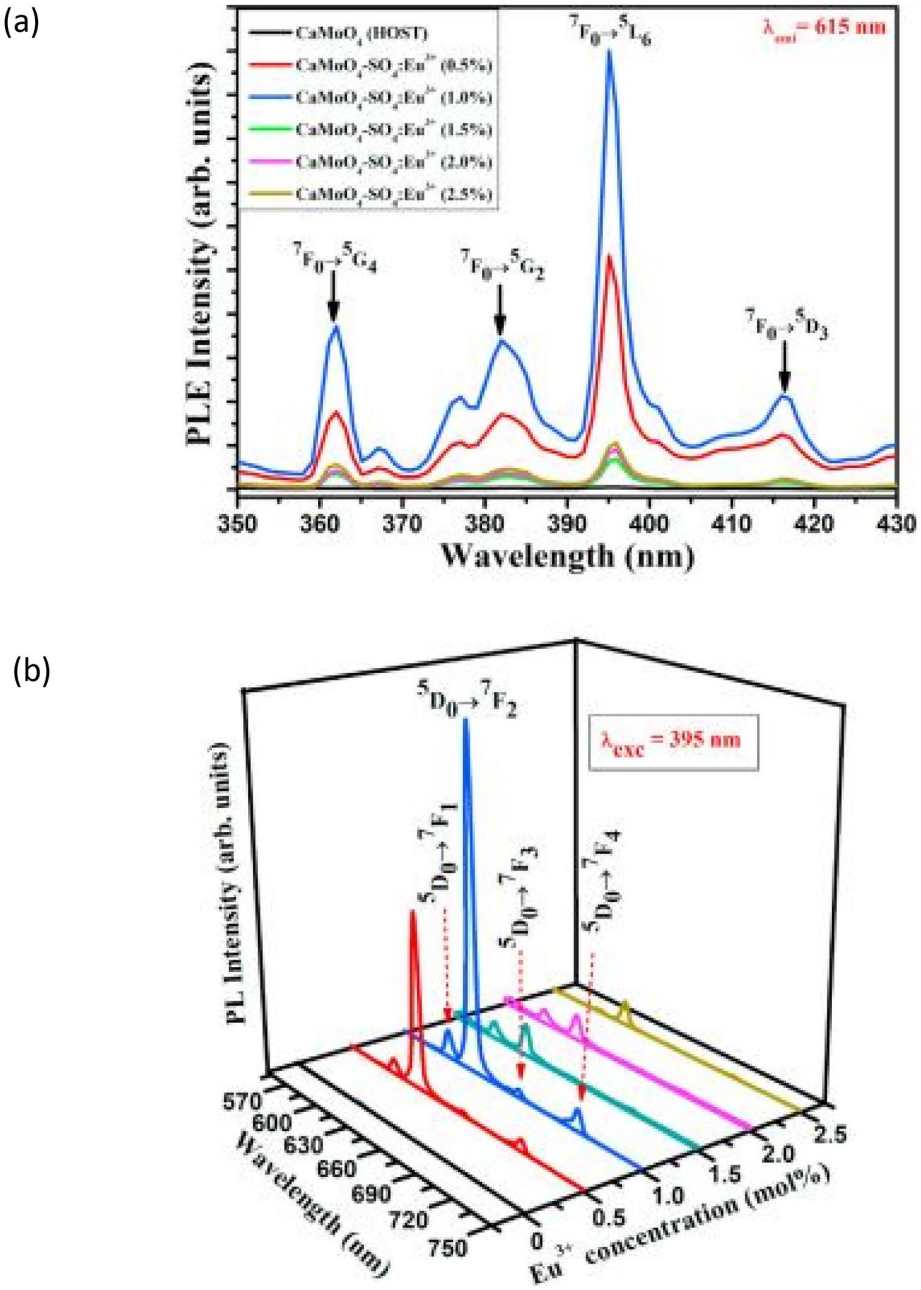
PLE spectra of the CaMoO_4_-SO_4_-xEu^3+^ phosphor material is shown in (**a**), while the PL emission spectra of the sample for concentration variations is shown in (**b**) [17] (Reused with permission from [17])

The PLE spectra of the CaMoO_4_-SO_4_:xEu^3+^ (x = 0.5 to 2.5 mol%) phosphor material, is measured in a wavelength range from 350 to 430 nm, as displayed above. The PLE spectra shows various excitation peaks, which are attributed to f-f transitions of Eu^3+^ ion from its ground state to various higher excited states. The maximum excitation peak occurs for a concentration of x = 1 mol% Eu^3+^ ion. The PL emission spectra is also shown above. The brightest red emission is produced again for a concentration of x = 1 mol%Eu^3+^ ion. It is observed that when SO_4_^2−^ ion was substituted into the host matrix, it caused reductions in the defective densities as non-radiative centers, which then led to improvements in distortions of the crystal fields that surrounds the Eu^3+^ dopant ion. The net effect of this is an effective energy transfer from the host CaMoO_4_ material to the dopant Eu^3+^ ion [18] (see Fig. 6).

### Important Finding of this Research

The interaction of the SO_4_^2−^ ions with Eu^3+^ ions creates crystal fields that influences the 4f electronic transitions of Eu^3+^ ions. In this scenario, the sensitizing effect of the integral SO_4_^2−^ ions is brought about by the absorption of energy and the transfer of that energy to Eu^3+^ ions. This and the combination of the crystal field effects, results in enhanced red colour emissions, for optimized concentration of x = 1 mol%. This confirms the fact that the anion SO_4_^2−^ is an excellent sensitizer for the CaMoO_4_ host material. If PL measurements were done with (non sulphates) the CaMoO_4_:xEu^3+^ phosphor materials and if further PL measurements were done after adding SO_4_^2−^, then the exact role of SO_4_^2−^ additions would have been clearly realized.

## Role of the Single Negative Phosphate Anionic Group (PO_4_^3−^) in the Sodium Orthophosphate Host NaMPO_4_:Ce^3+^ (M^2+^ = Mg, Ca, Sr, and Ba) Material Synthesized By Combustion Method [18]

The anionic phosphate PO_4_^3−^ group, which is an integral part of the Ce^3+^ activated orthophosphate NaMPO_4_ (M^2+^ = Ba, Ca, Sr, Mg) phosphor material, plays a crucial role in facilitating the energy transfer to the luminescecent centers within the material. The role of alkaline earth ions (cations) together with the anions are responsible for fine tuning the emission properties of the host material, thereby contributing to the overall photoluminescence behavior of the phosphor material.

### Review of the Key Features of this Study

**Fig. 7:**
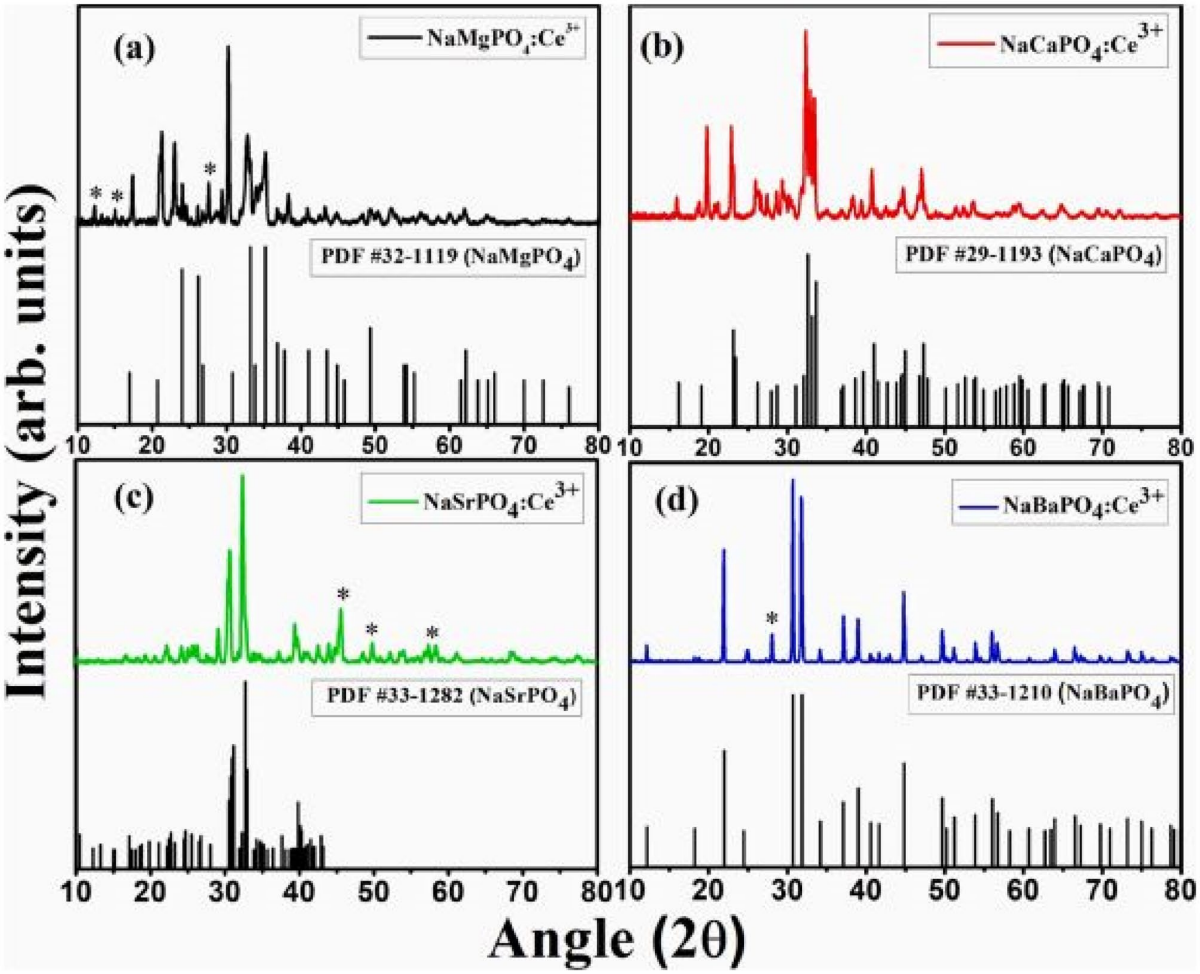
XRD patterns of the NaMPO_4_:Ce^3+^ (M^2+^ = Mg, Ca, Sr, and Ba) phosphor materials [19] (Reused with permission from [19])

XRD results by the combustion method, as shown in Fig. 7, likewise shows that the NaCaPO_4_ and NaBaPO_4_ phosphor materials are crystalized into orthorhombic phase structures, while the NaMgPO_4_ and NaSrPO_4_ phosphor materials are crystalized into monoclinic phase structures. The addition of the various alkaline earth ions were responsible for tuning of the crystal structures and, further the addition of the Ce^3+^ dopant to replace Na^+^/M^2+^ ions showed minimal changes in the diffraction patterns per transformed crystal structures. However, impurities were also observed due to incomplete dissolution of precursor molecules.

The deviation of the percentage error at lattice sites is obtained by using the same formula earlier in the section. The ionic radius of the following ions are: Ca^2+^ (1.00 Å), Mg^2+^(0.72 Å), Sr^2+^(1.18 Å), Ba^2+^(1.35 Å) and Ce^3+^(1.01 Å). This will give percentages of 1.00%, 40.27%, 14.41% and 25.19% for Ca^2+^, Mg^2+^, Sr^2+^ and Ba^2+^ ions when respectively replaced by the dopant. If CN = 8, then ${\Delta }_{r}$ would give values of 2.05%, 28.43%, 10.39% and 19.50% for Ca^2+^, Mg^2+^, Sr^2+^ and Ba^2+^ ions when respectively replaced by the dopant. Thus, replacing Ce^3+^ with Ca^2+^, Sr^2+^ and Ba^2+^ ions is more preferred, as it leads to minimal crystal distortions. Substitutions of Ce^3+^ ions with Mg^2+^ ions may cause slight distortions when CN = 6. It's also possible that additional peaks in the XRD graphs could result from mismatches in the ionic replacements or defects in the sample.

**Fig. 8:**
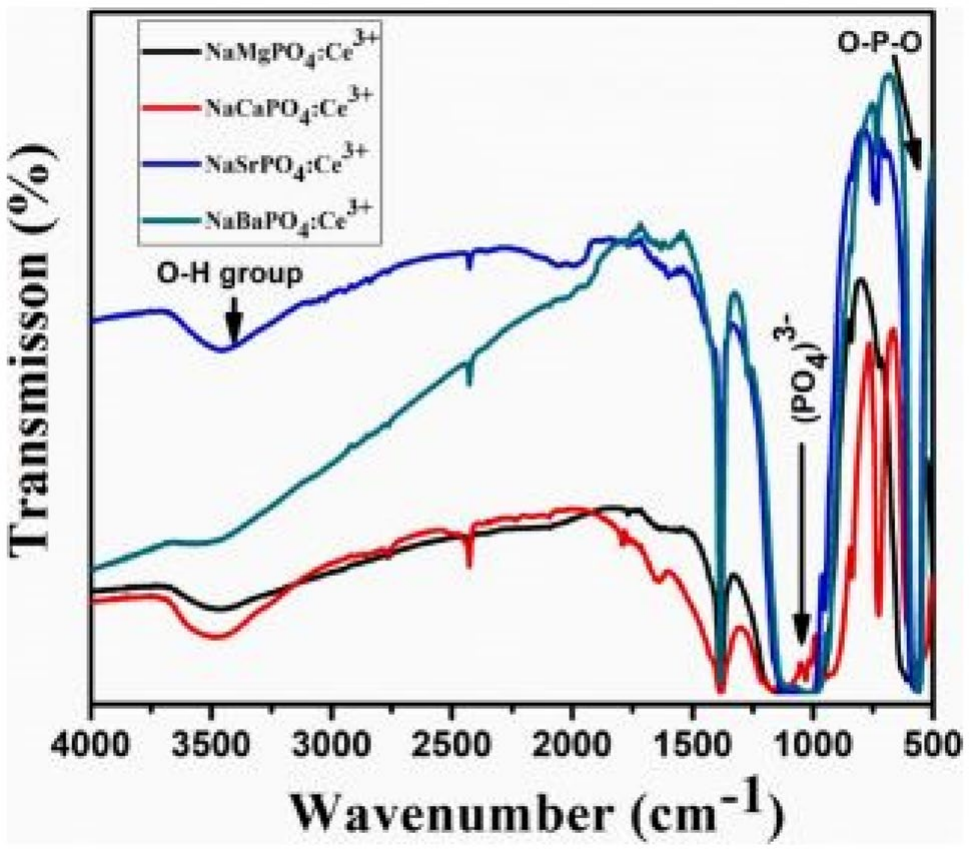
FTIR spectra of the NaMPO_4_:Ce^3+^ (M^2+^ = Mg, Ca, Sr, and Ba) phosphor materials [19] (Reused with permission from [19])

Figure 8 shows the FTIR measurements with the presence of O–P–O asymmetric bending vibrational modes, as well as the asymmetric stretching vibrations of the PO_4_^3−^ phosphate groups. The presence of these respective groups account for enhanced absorption properties of the phosphor materials. Other parameters, such as the OH groups could reduce the PL intensities.

**Fig. 9:**
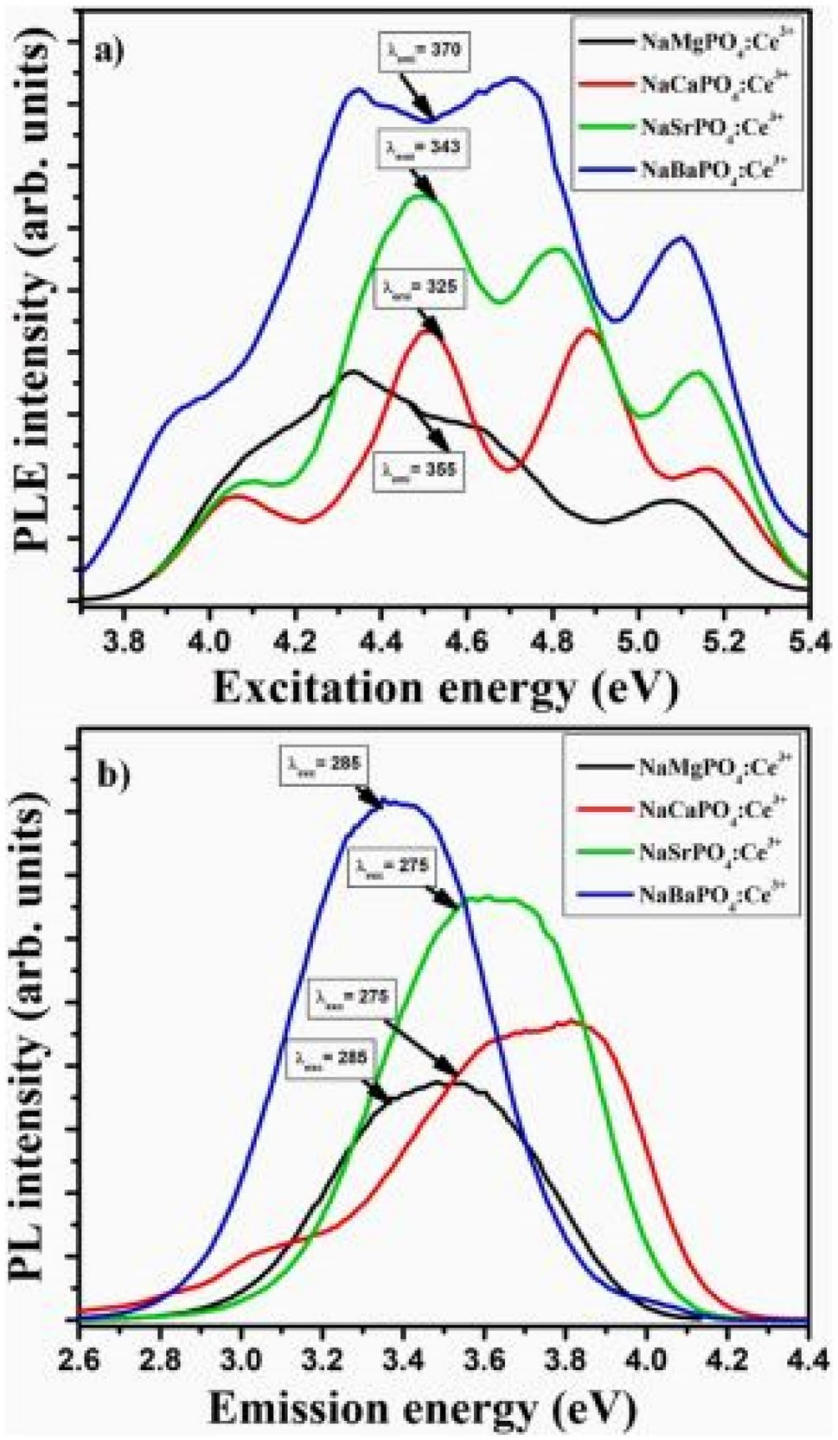
PLE excitation spectra for NaMPO_4_:Ce^3+^ (M^2+^ = Mg, Ca, Sr, and Ba) phosphor materials are shown in (**a**), while the PL emission spectra is shown in (**b**) as a function of binding energy for different wavelengths [19] (Reused with permission from [19])

The PLE excitation and PL emission spectra of the NaMPO_4_:Ce^3+^ phosphor materials are shown in Fig. 9 below. The excitation spectra consists of several peaks which are attributed to transitions 4d → 5d transitions of Ce^3+^ ions. The presence of doublet states is due to spin–orbit coupling of the 4f configurational state of Ce^3+^ ions. On the other hand, the PL emission spectra reveals emissions that arises from the split crystal field 5d excited state to the 4f spin- orbit coupled configurational state. Most enhanced emission in the UV-blue region is observed for the NaMgPO_4_-Ce^3+^ phosphor material compared to others, following a monoclinic crystallization, with Mg^2+^ cation component producing the best UV emission. It further appears that there are shifts in the emission properties of this material, with a tendency towards visible emission when Ce^3+^ dopant is used. In this scenario, the role of the phosphate PO_4_^3−^ (inherent) group as a sensitizer is to absorb energy from the excitation source and transfer it non-radiatively to Ce^3+^ ions. The presence of Ca^2+^ and Ba^2+^ ions, in the combustion method at 600 °C, and their arrangement with the phosphate ions (PO_4_^3−^) in the crystal lattice, favours the creation of orthorhombic crystal structure, while Mg^2+^ and Sr^2+^ ions favors monoclinic structures (see Fig. 9).

### Important Finding of this Research

The presence of the PO_4_^3−^group in the NaMPO_4_ phosphor material play important functions. Firstly, it creates a crystal field when it interacts with the positive charges. Secondly, it acts as sensitizers in absorbing excitation energy and transferring it non-radiatively to the Ce^3+^dopants. Thirdly, it creates a stable crystal structure for Ce^3+^ dopant embedding. These factors contribute to enhanced UV-blue emissions for this phosphor material, synthesized by the combustion method of synthesis, and causes shifts in the emission spectra due to cationic tuning. Such emissions are useful for the development of advanced LED and technological device systems.

## Role of the Single Negative Anionic Phosphate Group (PO_4_^3−^) in the Calcium Phosphate Host Ca_3_(PO_4_)_2_: Ce^3+^ and Co-doped with Gd^3+^ Ions [20]

The inherent presence of the phosphate PO_4_^3−^ group in the calcium phosphate Ca_3_(PO_4_)_2_ host material, doped with violet emitting Ce^3+^ and co-doped with UV-B emitting Gd^3+^ ions, has a significant impact on the luminescence characteristics when synthesized by the combustion synthesis method. The role of the phosphate group, coupled with the dopant ions plays an important role in shifting the emission wavelengths of Ce^3+^ ions when co-doped with Gd^3+^ ions. No FTIR measurements were done for this research.

### Review of the Key Features of this Study

**Fig. 10:**
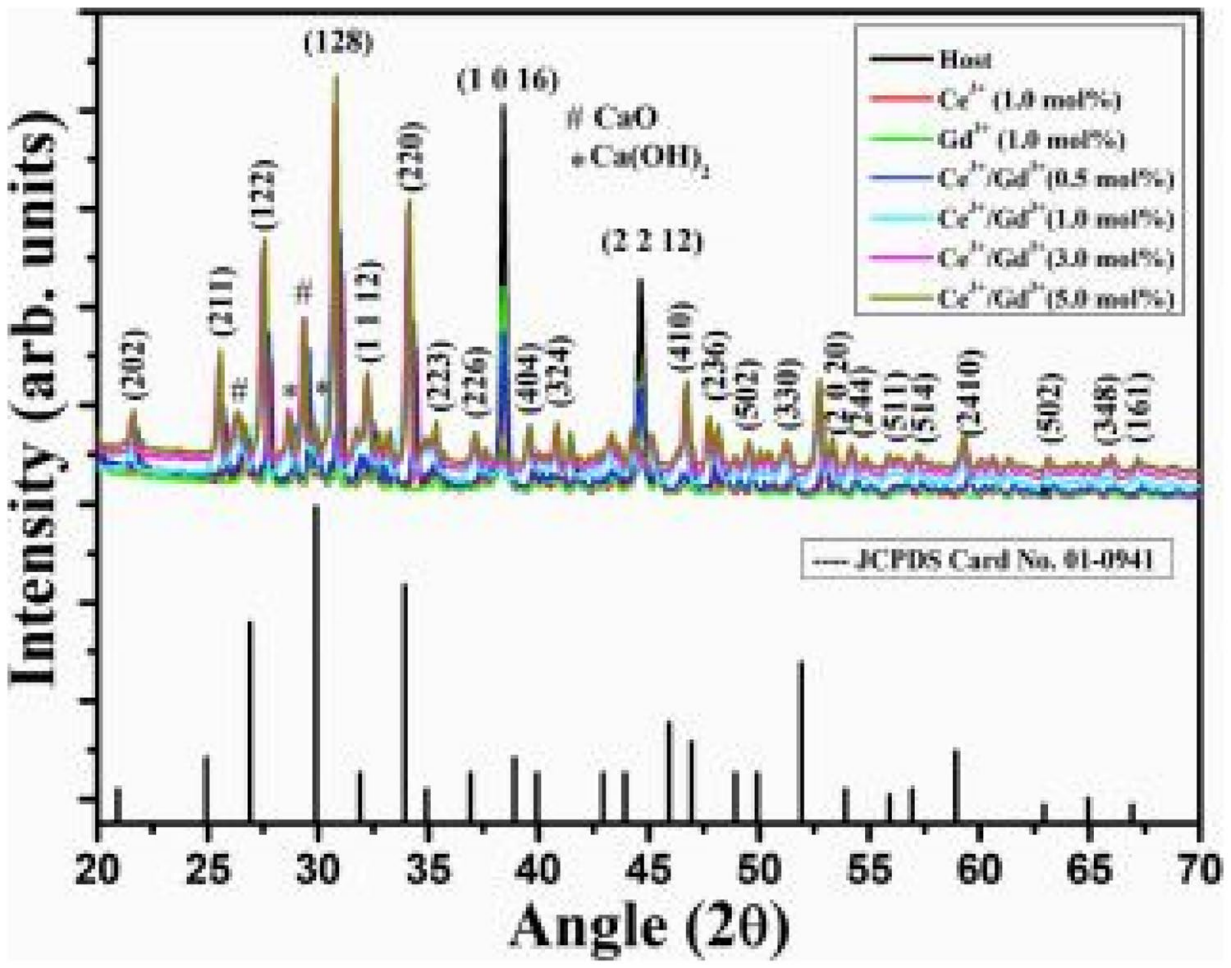
XRD patterns of the Ca_3_(PO_4_)_2_: Ce^3+^phosphor material co-doped with Gd^3+^ ions [20] (Reused with permission from [20])

The XRD in Fig. 10 shows the formation of a rhombohedral phase structure, according to the JCPDS file structure classification. Complete transformation to pure β-Ca_3_(PO_4_)_2_ structure was achieved when calcined to a temperature of 900^0^C and above. The replacement of Ca^2+^ ions by Gd^3+^ ions (for increases in concentration) shifts the diffraction peaks to lower 2θ angles, implying an expansion of the crystal lattice structure. Additions of the Ce^3+^ ions to the host material leaves the rhombohedral crystal structure unchanged, thus conforming to crystallinity. Impurities such as CaO and Ca(OH)_2_ were also formed in the experimental process, except at high concentrations of Gd^3+^ ions.

**Fig. 11:**
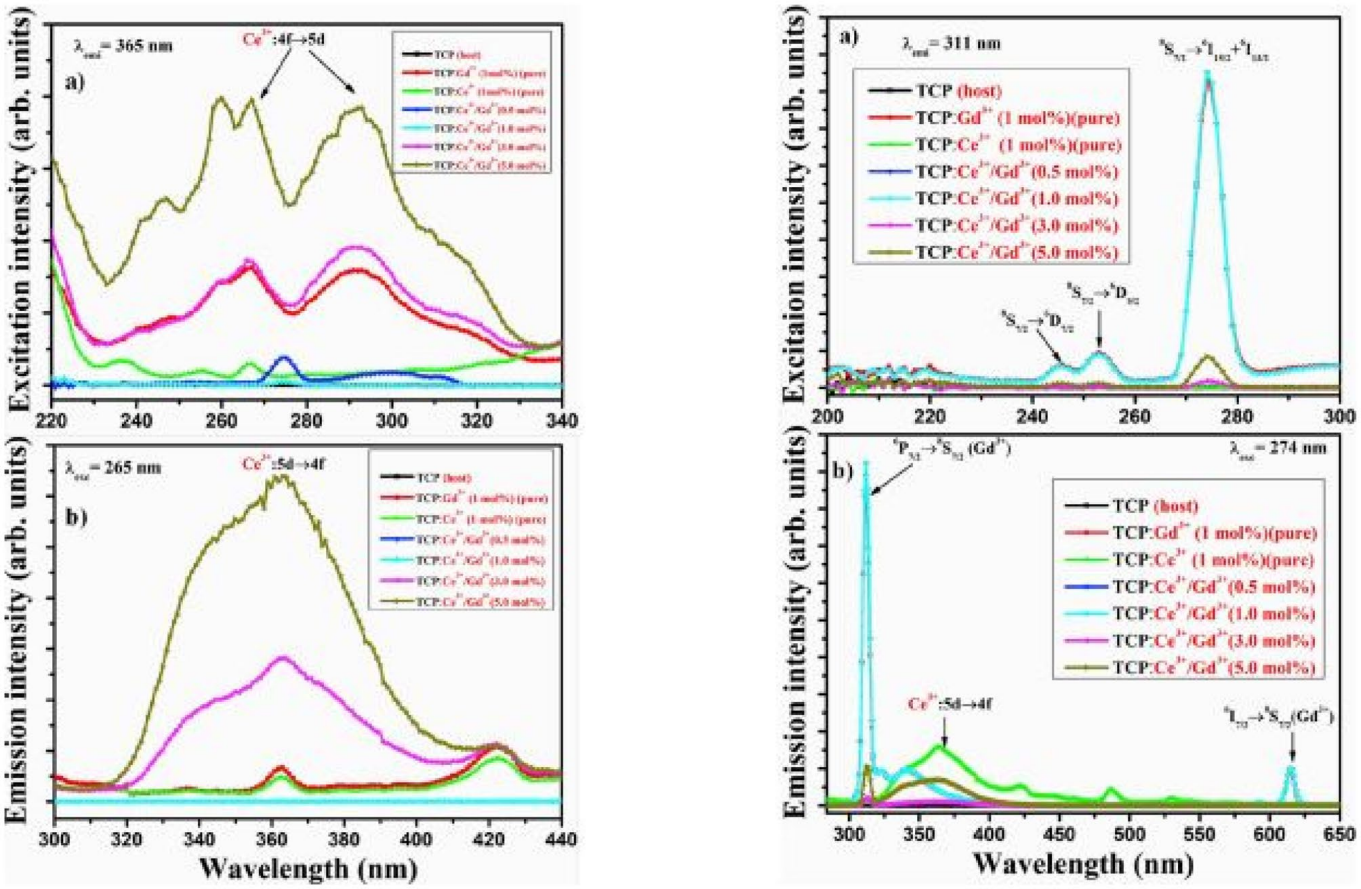
PLE excitation and PL emission spectra of Gd^3+^ co-doped Ca_3_(PO_4_)_2_: Ce^3+^phosphor material, excited at different wavelengths in respective pairs at (**a**) and (**b**) [20] (Reused with permission from [20])

The deviation percentage error at lattice sites are obtained using the same formula earlier in the section. For this section, ${\Delta }_{r}$ has a percentage error of 1.00% and 6.20% for CN = 6 when Ce^3+^ or Gd^3+^ replaces Ca^2+^ ions, and a percentage error of 2.05% and 5.98% for CN = 8, when Ce^3+^ or Gd^3+^ replaces Ca^2+^ ions. Since the absolute values are far less than 30%, implies excellent replacements. The additions of the phosphate group, if sitting at interstitial positions could cause additional peaks in the XRD diffraction patterns (see Fig. 11).

The PLE excitation and PL emission spectra of the Ca_3_(PO_4_)_2_: Ce^3+^/xGd^3+^ phosphor materials are shown in the figures below. The PLE excitation spectra consists of several excitation peaks which are attributed to 4f → 5d transitions of Ce^3+^ ions in the wavelength range of 220 nm to 440 nm. Its PL emission spectra on the other hand consists of a broad UV emission peak, centered around 365 nm when monitored at a wavelength of 265 nm. This dynamic changes drastically if the co-doped samples are excited using an excitation wavelength of 274 nm, whilst monitored at an emission wavelength of 311 nm. In one scenario, we observe violet-blue emissions due to Ce^3+^ ions alone, but in another scenario, we observe both emissions by Gd^3+^ (major UV-B emission at 311 nm) and another by Ce^3+^ (minor emission in the violet-blue region at 365 nm), which is dependent on the excitation wavelength chosen for the emission properties. The role of doping with Gd^3+^ ions was to shift the Ce^3+^ emission to the red region (611 nm) for a concentration of 1 mol%, which is crucial for white light emissions. Likewise, as with other materials, the inherent presence of the phosphate (PO_4_^3−^) ions creates a stable environment for dopant embedding and aids in the sensitization effect by transferring the absorbed energies to the dopant ions, thereby producing enhanced emissions.

### Important Findings of the Study

The role of the phosphate PO_4_^3−^ group is very fascinating in this scenario. It creates crystal fields that shifts the energy peaks of the emission curves, forms stable structures for dopant embedding, and serves as a partial sensitizer alongside Gd^3+^ ions in absorbing excitation energy and transferring it to Ce^3+^ ions, thereby producing enhanced emissions. Depending on the excitation wavelength, either the violet-blue emissions due to Ce^3+^ are enhanced or UV-B emissions due to Gd^3+^ ions are enhanced. This implies that the energy transfer process can be bi-directional. These emissions produced can find applications in phototherapy or in scintillating devices. Interestingly, red emissions due to Ce^3+^ ions are observed for a concentration of 1 mol% Gd^3+^ ions, besides the signature violet-blue emissions due to Ce^3+^ ions.

## Role of the Single Negative Sulphate Ion Anionic Group (SO_4_^2−^) Substitution in Zinc Oxide Host ZnO: Ce^3+^ Phosphor Materials [21]

In this scenario, the sulphate SO_4_^2−^ group serves as a substituent, which acts as a sensitizer to Ce^3+^ activated ZnO phosphor host material, synthesized using the solid-state reaction method. The crucial role the SO_4_^2−^ anions play in this defect induced PL emissions of ZnO phosphor materials will be explored. The role of Ce^3+^ ions substitution in the host material is also of crucial importance in understanding the luminescence characteristics of this phosphor material.

### Review of the Key Features of this Study

The XRD patterns of ZnO, ZnO-SO_4_ and ZnO-SO_4_:xCe^3+^ (x = 0.5 to 2.5 mol%) phosphor materials are shown in the figure above. All XRD diffraction patterns conform to a hexagonal wurtzite structure of pure ZnO phosphor materials. Additions of SO_4_^2−^ results in no additional peaks, implying good dissolution of the ZnO host matrix. This stems from the fact that the anion SO_4_^2−^ of ionic radius 0.147 nm is replacing an oxygen O^2−^ ion of similar ionic radius of 0.140 nm. However, the addition of SO_4_^2−^ shifts the diffraction peaks to the right of 2θ (contraction), while the addition of Ce^3+^ shifts the diffraction peaks to lower 2θ values (expansion). Impurities, which originates from Ce^3+^ doping were observed in this spectrum as well.

**Fig. 12:**
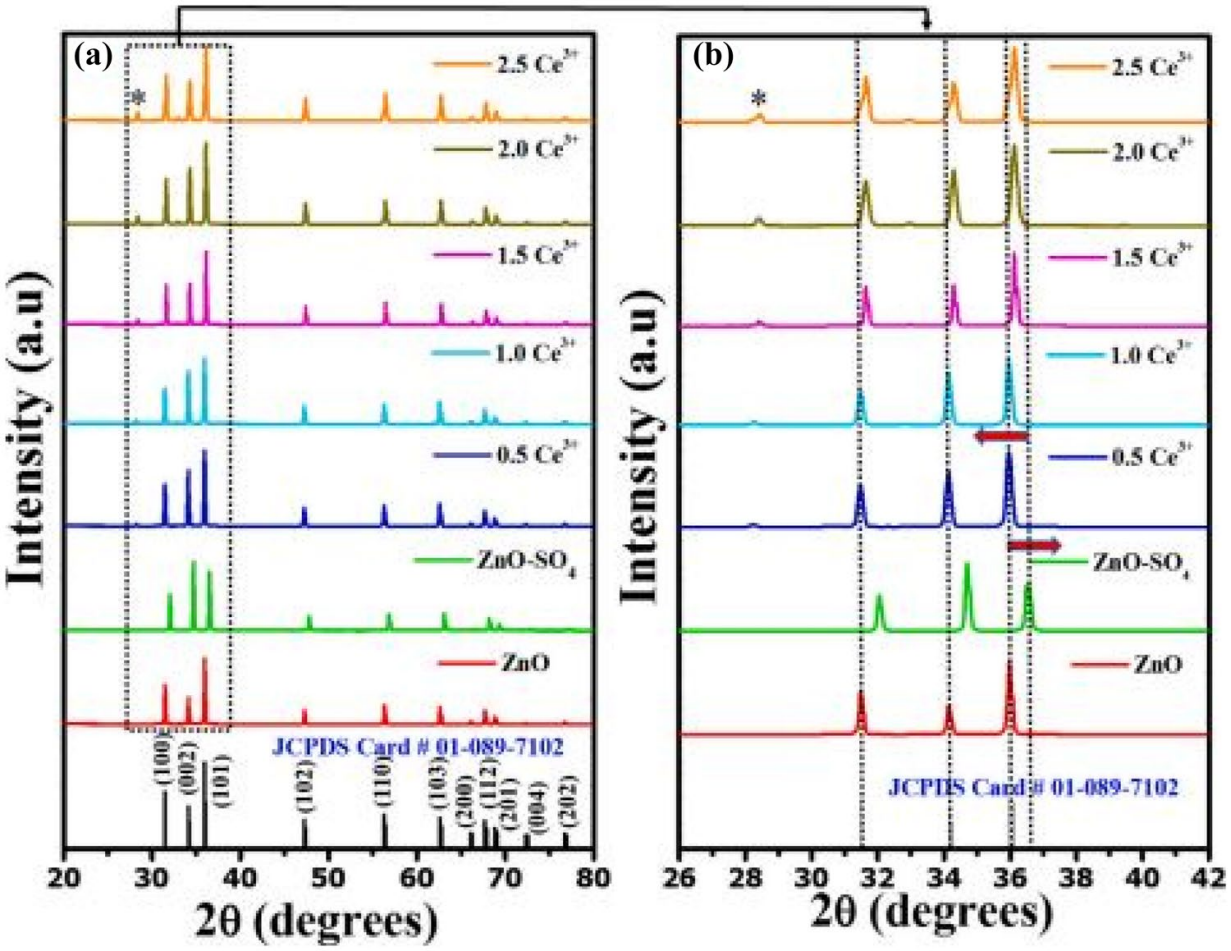
XRD patterns of the ZnO: Ce^3+^phosphor material with SO_4_^2-^ anionic incorporation in (**a**) and enlarged section in (**b**) showing the relative shifts in diffraction peaks [21] (Reused with permission from [21])

The deviation in percentage error at lattice sites are obtained using the formula earlier in the section. For this section, ${\Delta }_{r}$ has a percentage error of 26.73% when CN = 6 and a percentage error of 21.26% when CN = 8 when Ce^3+^ ion replaces Zn^2+^ ions in the crystal lattice (see Fig. 12).

**Fig. 13:**
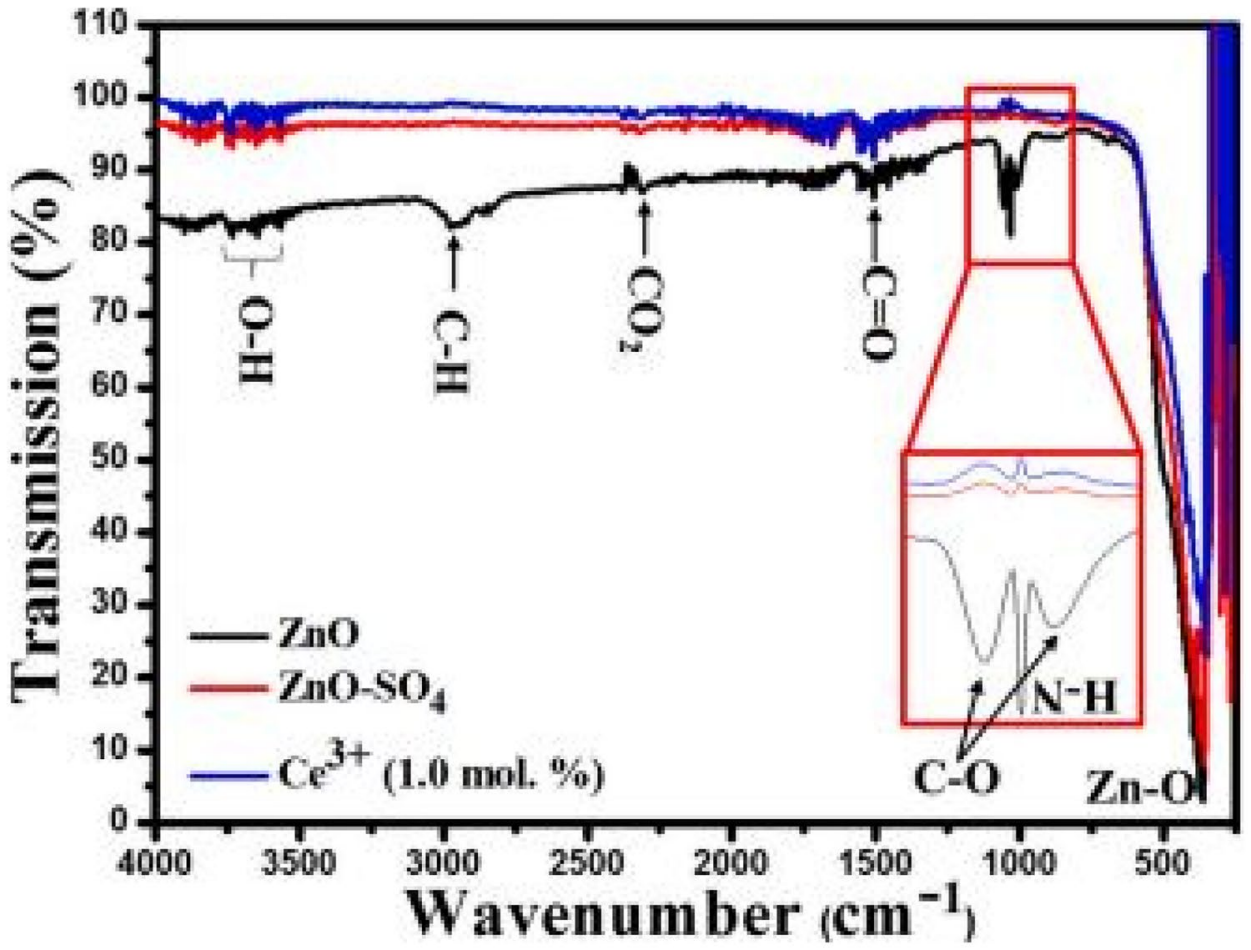
FTIR spectra of the ZnO: Ce^3+^ phosphor materials with SO_4_^2−^ incorporation [21] (Reused with permission from [21])

The FTIR measurements of the ZnO: Ce^3+^ phosphor materials with SO_4_^2−^ incorporation is shown in Fig. 13 above. This quite clearly show shifts in FTIR absorption behavior for SO_4_^2−^ additions and M–O bonded structures for wavenumbers below 500 cm^−1^ compared to the pure ZnO host material. Further, both Ce^3+^ ions and SO_4_^2−^ additions limit the absorptions of other unnecessary functional groups in the FTIR spectra. This will help in observing specific features in the absorption spectra of the ZnO: Ce^3+^-SO_4_ phosphor materials.

**Fig. 14:**
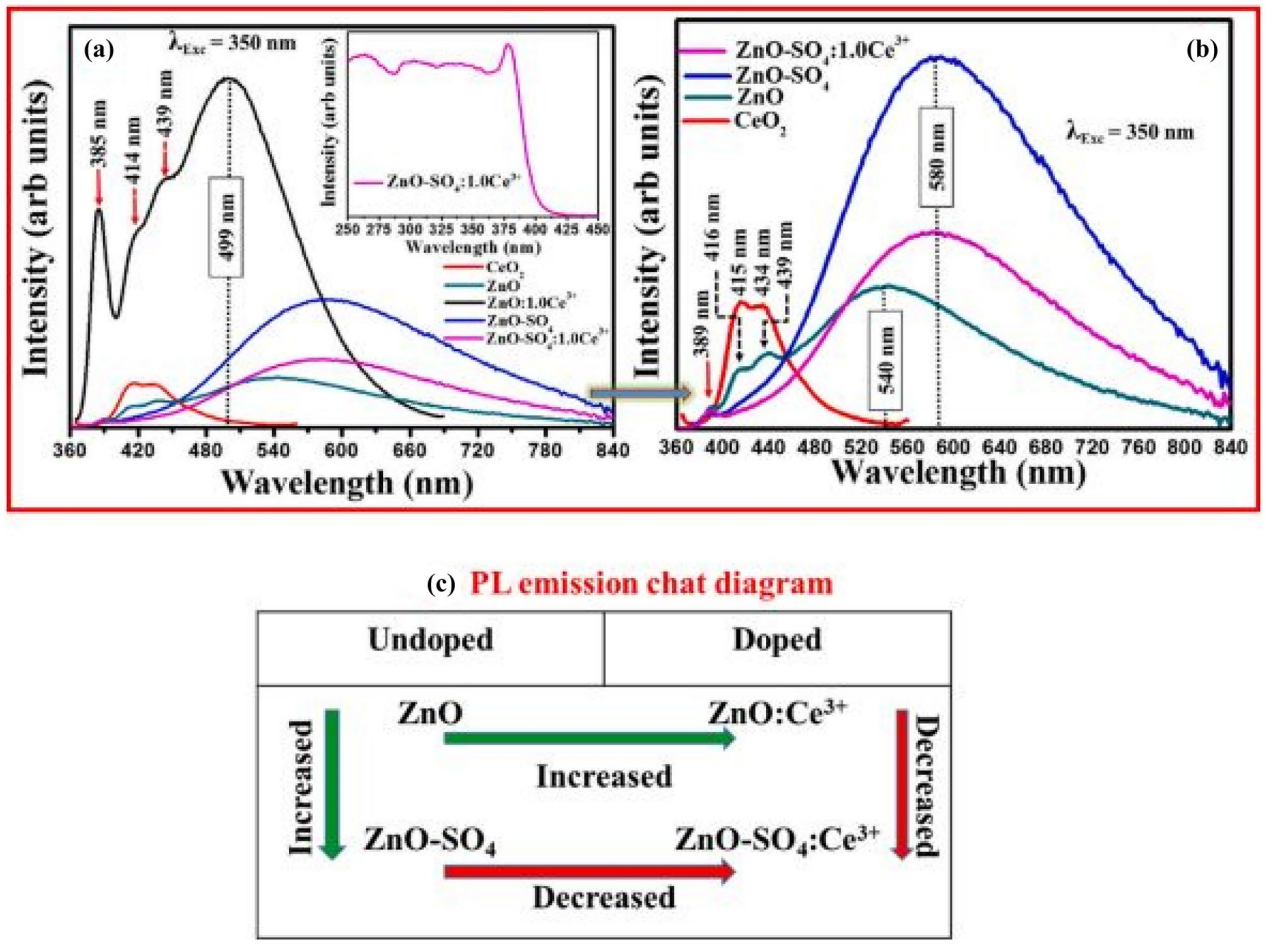
PL emission spectra of the ZnO: Ce^3+^ phosphor materials, with SO_4_^2-^ incorporations in (**a**), and (**b**) showing enlarged sections of (**a**), and (**c**) shows the trends of PL emission when SO_4_^2-^ or Ce^3+^ ions are added to ZnO [21] (Reused with permission from [21])

Figure 14 shows the PL emission spectra of the ZnO: Ce^3+^ phosphor materials with SO_4_^2−^ incorporation, in a wavelength range from 360 to 840 nm. The role of SO_4_^2−^ here is remarkable in enhancing the photoluminescence properties of the phosphor material, high intensity (many folds higher) PL emission is observed when SO_4_^2−^ ions are added to ZnO phosphor materials. Thus SO_4_^2−^ effectively absorbs the excitation energy and transfers it non-radiatively to ZnO material. Emissions are due to transitions from the doublet degenerate 5d level of Ce^3+^ ions to its spin–orbit coupling of the 4f configurational state of Ce^3+^ ion. On the other hand, the role of Ce^3+^ ions is one to quench the PL luminescence by taking energy away from the luminescent centers. Furthermore, shifts towards red emissions, a requirement for white emission is observed through SO_4_^2−^ and Ce^3+^ additions.

### Important Findings of the Study

The introduction of sulfate ions SO_4_^2−^ can create specific defects (Zn^2+^/O^2−^) within the band gap of ZnO host lattice, which act as emission centers themselves (defect emissions). These defective emission centers, due to the creation of various vacancy sites within the band gap of the host can lead to characteristic colour emissions in the nanophosphors, contributing to their unique luminescence behavior. The role of sulfate ions SO_4_^2−^ is to introduce defective sites, and these can provide color tunability in the emission of ZnO: Ce^3+^ phosphor materials. These distinctive defective colour emissions allows for precise control of the emission wavelengths, which is important in color display technology to achieve various color outputs.

The sulphate ions (SO_4_^2−^) play a crucial role in this material as a substituent. Firstly, it creates crystal fields that drastically affects the electronic transitions of the Ce^3+^ ions and their emission properties. Secondly, it acts as a sensitizer in absorbing excitation energy and transferring it to ZnO, which causes huge emission effects. Thirdly, they are responsible for the creation of defects in the host material, for defective emissions. Functionally, we observe the following, an increase in PL emission when SO_4_^2−^ are present, but a decrease in PL intensity when Ce^3+^ ions are added. This is due to reductions in radiative recombination caused by Ce^3+^ absorbing energy and transferring it radiatively away from their luminescent centers.

## Role of the Single Negative Borate Group (BO_3_^3−^) in the Calcium Molybdate Host CaMoO_4_:Eu^3+^ Phosphor Material [22]

Borate ions (BO_3_^3−^) act as modifiers in the host matrix of the Eu^3+^ activated CaMoO_4_ phosphor material. The substitution of borate ions (BO_3_^3−^) for other ions, such as molybdate ions (MoO_4_^2−^), can modify the crystal structure and lattice parameters of the phosphor material. This modification can influence the electronic transitions and the local crystal field surrounding the luminescent red emitting Eu^3+^ dopant ions. The presence of these borate ions (BO_3_^3−^) creates crystal fields surrounding the Eu^3+^ dopant ions, leading to changes in their electronic transitions as well as to radiative recombination. Borate ions (BO_3_^3−^) can facilitate the efficient energy transfer to Eu^3+^ ions. This includes absorption of excitation energy and transferring it non-radiatively to Eu^3+^ ions or vice versa.

### Review of the Key Features of this Study

**Fig. 15:**
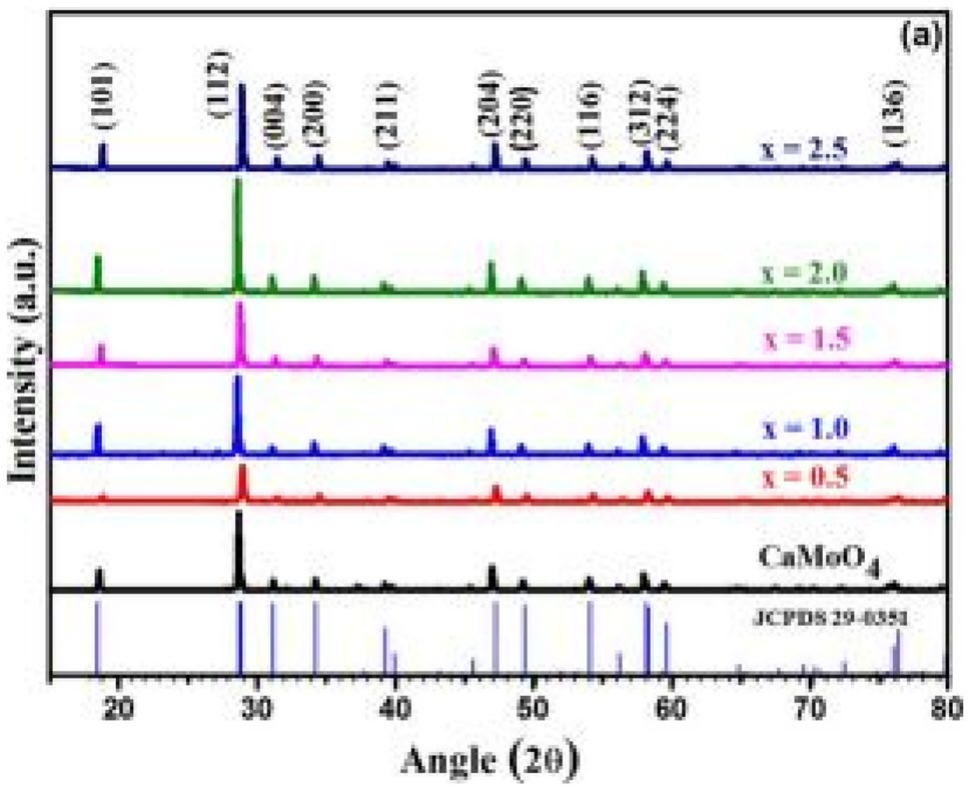
XRD spectra of the CaMoO_4_:Eu^3+^ phosphor material with BO_3_^3−^ incorporations [22] (Reused with permission from [22])

The XRD patterns of the Eu^3+^ activated calcium molybdate CaMoO_4_ host materials and further doping with BO_3_^3−^ incorporation, are shown in Fig. 15 below. The tetragonal structure of the host material is hardly affected by the incorporation of both Eu^3+^ (for Ca^2+^ ions) ions and the borate BO_3_^3−^ (for MoO_4_^2−^ ions) ions. However slight shift in the diffraction peaks were observed when BO_3_^3−^ ions were introduced into the host matrix, which led to minor structural changes.

The deviation in percentage error at lattice sites is obtained using the same formula earlier in the section. For this section, ${\Delta }_{r}$ has a percentage error of 5.3% for CN = 6 and a percentage error of 4.82% for CN = 8 when Ce^3+^ ions replaces Ca^2+^ ions at lattice positions. This value is likewise less than 30% and implies good replacement for Ca^2+^ ions with some crystal distortions. The additions of borate and dopants causes shifts in the diffraction patterns of Fig. 15.

**Fig. 16:**
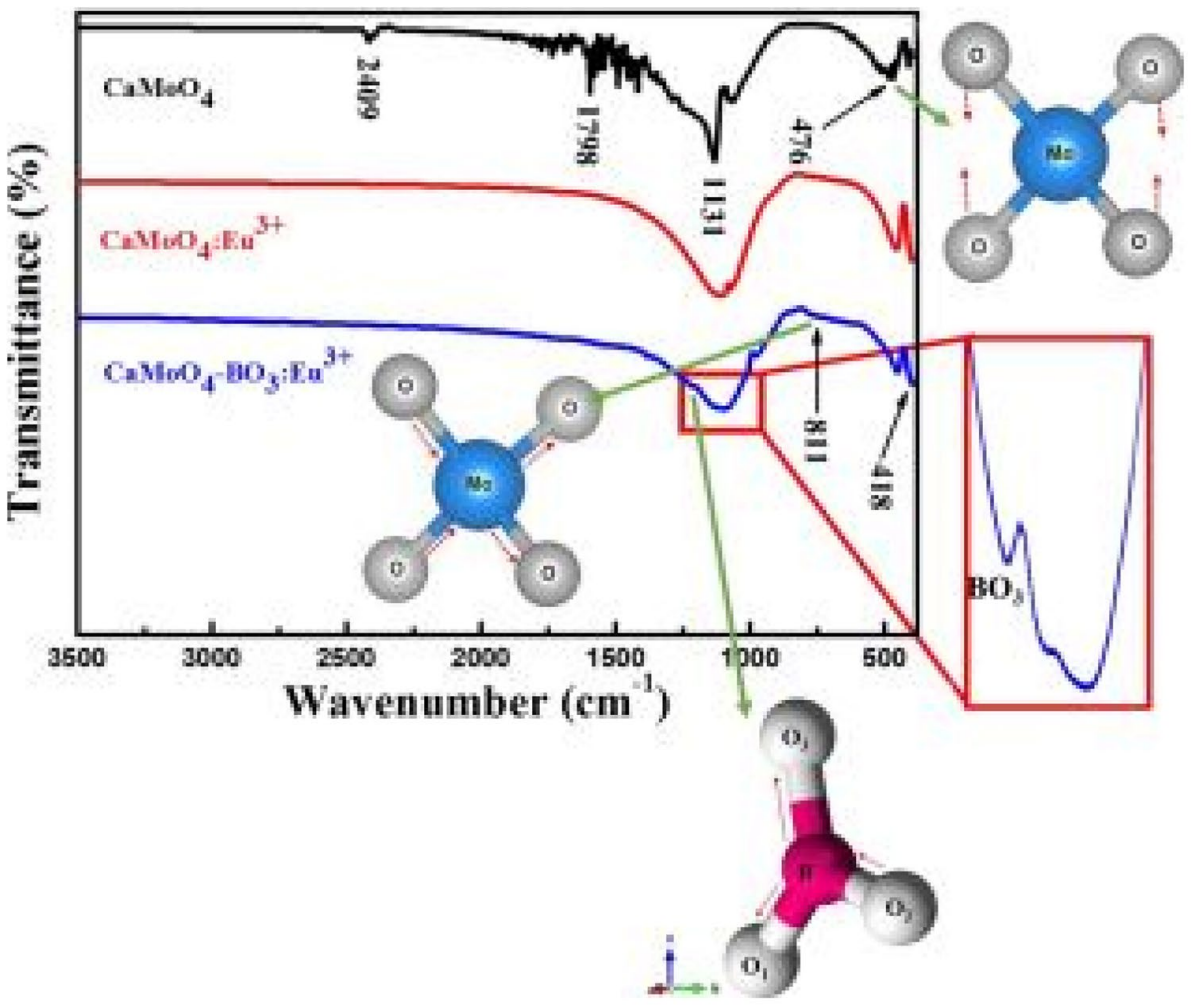
FTIR spectra of the CaMoO_4_:Eu^3+^ phosphor material with BO_3_^3-^ incorporation [22] (Reused with permission from [22])

The FTIR measurements, shown in Fig. 16 below, reveals absorption by the borate BO_3_^3−^ group is diminished when incorporated into the Eu^3+^ activated CaMoO_4_ host material, but with slight reductions in the absorption bands of unnecessary functional groups, thus useful in tailoring its electronic properties for useful absorptions.

**Fig. 17:**
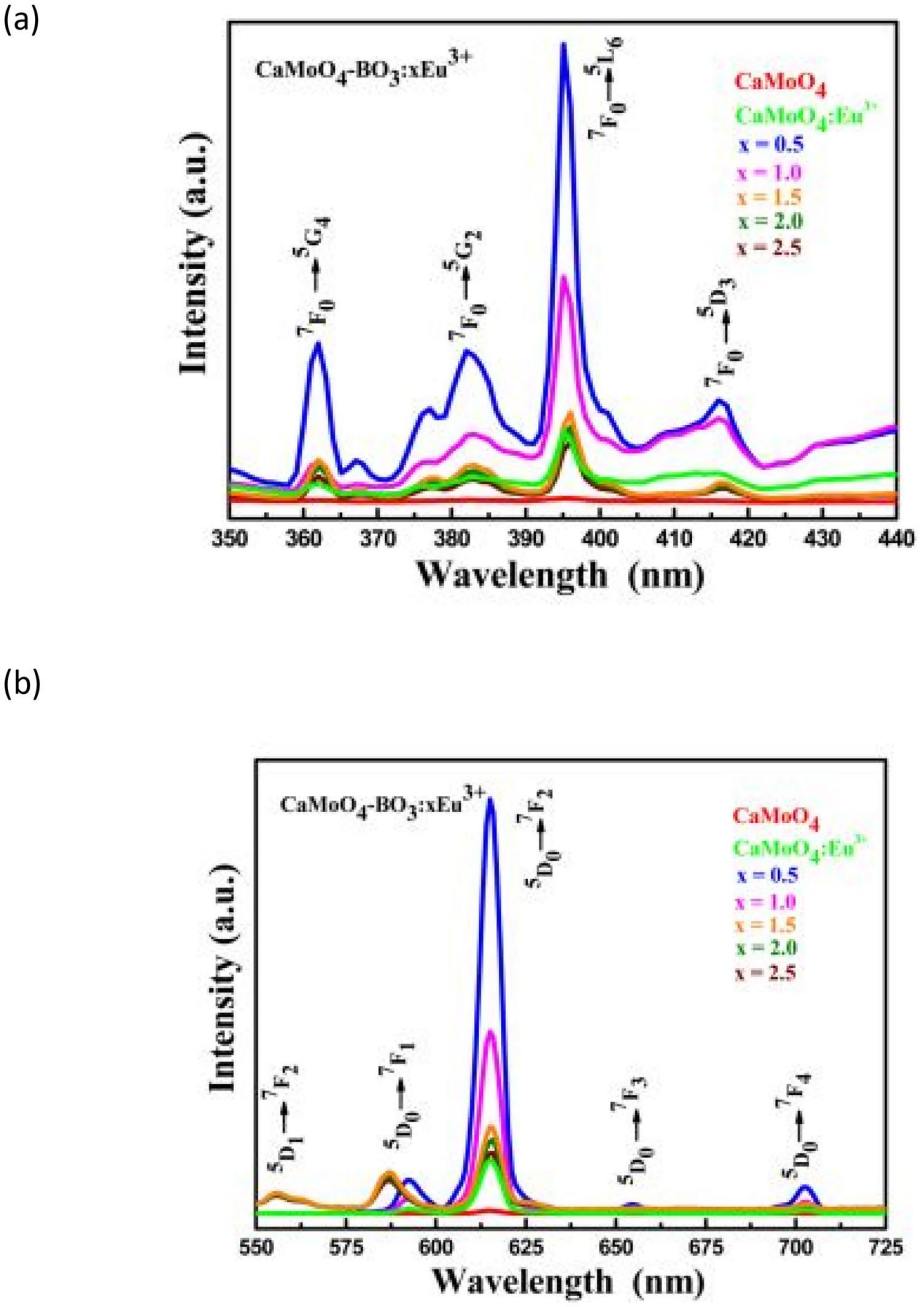
PLE excitation spectra of the CaMoO_4_:Eu^3+^ phosphor material is shown in (**a**), while the PL emission spectra of the same sample is shown in (**b**) with BO_3_^3-^ incorporations [22] (Reused with permission from [22])

Figure below shows the PLE excitation spectra of the CaMoO_4_-BO_3_:xEu^3+^ (x = 0 to 2.5 mol%) phosphor material, measured in a wavelength range from 350 to 440 nm. Various excitation peaks are observed which are attributed to the intraconfigurational f-f transitions of the Eu^3+^ ion in the host material, whilst moving from its ground state to various higher excited states. The highest excitation peak occurs at around 395 nm which corresponds to a concentration of x = 0.5 mol% Eu^3+^ ion. The PL emission spectra for the prepared phosphor material is shown alongside this diagram. Enhance red emissions are tuned with concentration variations of Eu^3+^ ion, with optimal red emissions occurring for x = 0.5 mol%. The presence of borate ions BO_3_^3−^ has enhanced the photoluminescence behavior of the CaMoO_4_:Eu^3+^ phosphor material, but not as efficiently as with SO_4_^2−^ or PO_4_^3−^ incorporations for high concentrations of Eu^3+^ ions. By comparing the emissions of the CaMoO_4_-BO_3_:xEu^3+^ and CaMoO_4_:Eu^3+^ (at 1 mol. % of Eu^3+^) phosphor materials, shows that the addition of the borate anionic groups (BO_3_^3−^) systems has effectively enhanced the PL intensity of CaMoO_4_:Eu^3+^ phosphor material. The borate ions BO_3_^3−^ may act as sensitizers, is useful, in absorbing the excitation energy and transferring it non-radiatively to the Eu^3+^ ions, which, in turn, results in increased luminescent characteristics (see Fig. 17).

### Important Findings of this Study

In the CaMoO_4_:Eu^3+^ phosphor material, borate group (BO_3_^3−^) plays a crucial sensitizing role in influencing the local crystal field surrounding the Eu^3+^ dopant ions for low concentrations. It actively participates in the energy transfer processes, thereby enhancing the photoluminescence characteristics of the phosphor material. These research findings contribute a better understanding of BO_3_^3−^ doped phosphor material, when compared to PO_4_^3−^ and SO_4_^2−^ ions, for the development of advanced optoelectronic devices for LED applications.

## Role of Anions PO_4_^3−^, SO_4_^2−^ and BO_3_^3−^ in the Yttrium Oxide Host Y_2_O_3_:Eu^3+^ Phosphor Material [23]

The primary objective of this study is to understand how the incorporation of different anions ([BO_3_^3−^], [PO_4_^3−^], and [SO_4_^2−^]) will influence the structural and red emitting properties of the Y_2_O_3_:Eu^3+^ phosphor materials. More specifically, this research intends to evaluate how the presence of these anions influences the emission characteristics of the phosphor material. The key focus of this research is on the photoluminescence properties of the Y_2_O_3_:Eu^3+^ phosphors. The Eu^3+^ ion is a well-known red-emitting rare earth dopant, and its emission intensity and spectral features are sensitive to the local crystal field environment, surrounding the dopant ions and the presence of the anions. Due to transformation of crystal structures, the emission must be studied individually.

### Review of the Key Features of this Study

**Fig. 18:**
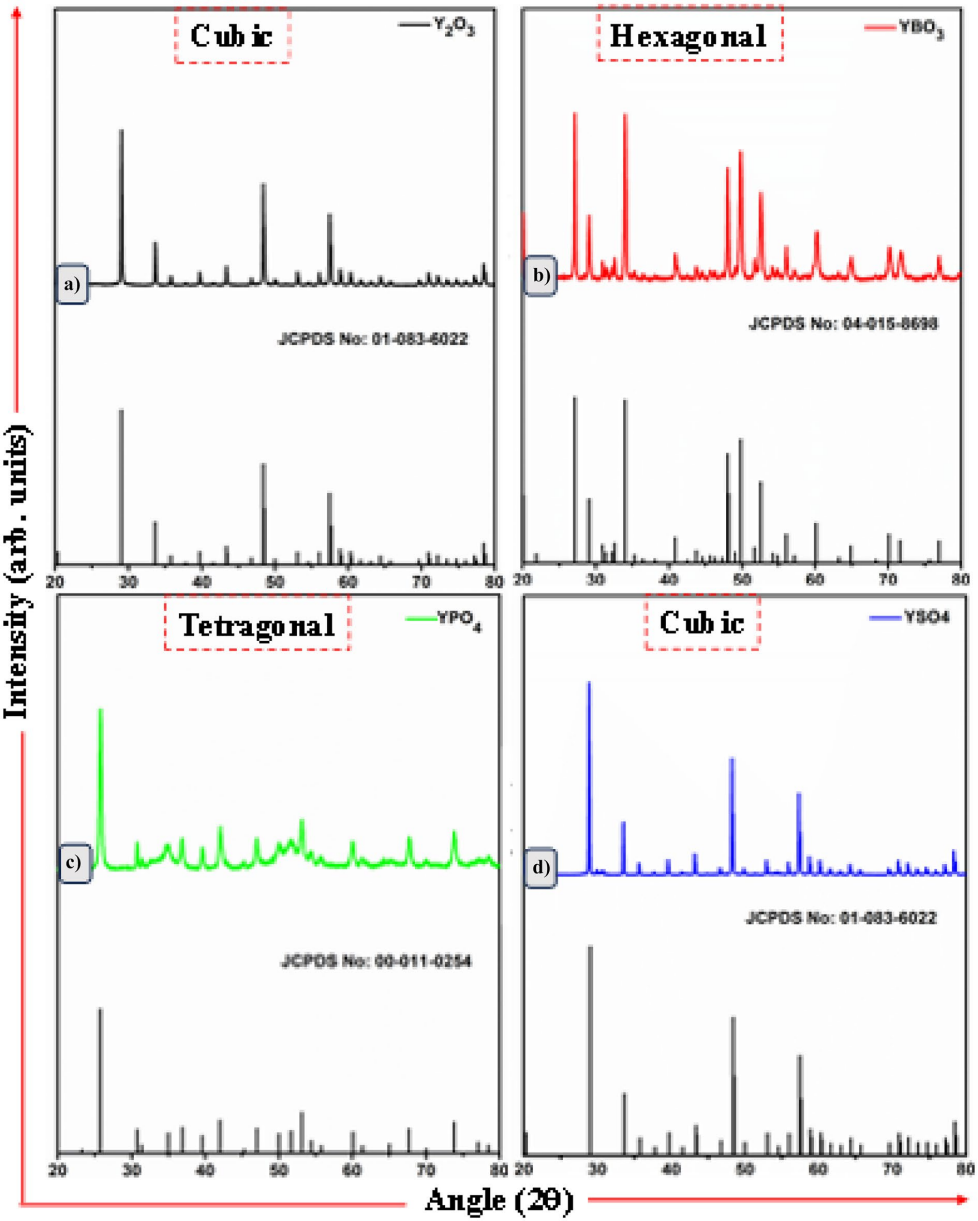
XRD pattern of the Y_2_O_3_-AG-Eu^3+^ phosphor materials, with AG = (BO_3_^3−^), (PO_4_^3−^), and (SO_4_^2−^) additions, and transformed structures for borate and phosphate additions [23] (Reused with permission from [23])

Figure 18 shows the XRD spectra of the Y_2_O_3_-AG-Eu^3+^ (where AG represents the various anions) phosphor materials. It is observed that the anions were found to modify the crystal structure of the Y_2_O_3_ phosphor materials, producing a cubic crystal structure for Y_2_O_3_-SO_4_-Eu^3+^, a hexagonal crystal structure for Y_2_O_3_-BO_3_^3−^-Eu^3+^ and a tetragonal crystal structure for Y_2_O_3_-PO_4_^3−^ -Eu^3+^ phosphor materials. The addition of Eu^3+^ (1.17 Å) with a similar radius to Y^3+^ (1.011 Å) has had no significant change to the diffraction patterns of transformed crystal structures. However, the addition of anions has transformed the crystal structures, as mentioned above, with reductions in the unit cell volumes for borate and phosphate compounds. These unique crystal structure account for the luminescence properties of the phosphor materials, and it must be concluded which crystal structure produces the best red emissions.

The deviation in percentage error at lattice sites is obtained using the same formula earlier in the section. For this section, ${\Delta }_{r}$ has a percentage error of 4.96% for CN = 6 and a percentage error of 4.41% for CN = 8 when Y^3+^ ions are replaced by Eu^3+^ions in the crystal lattice. This value of less than 30% is good and this is also reflected in their XRD graphs by revealing minimal changes in the diffraction peak positions and intensities of transformed structures.

**Fig. 19:**
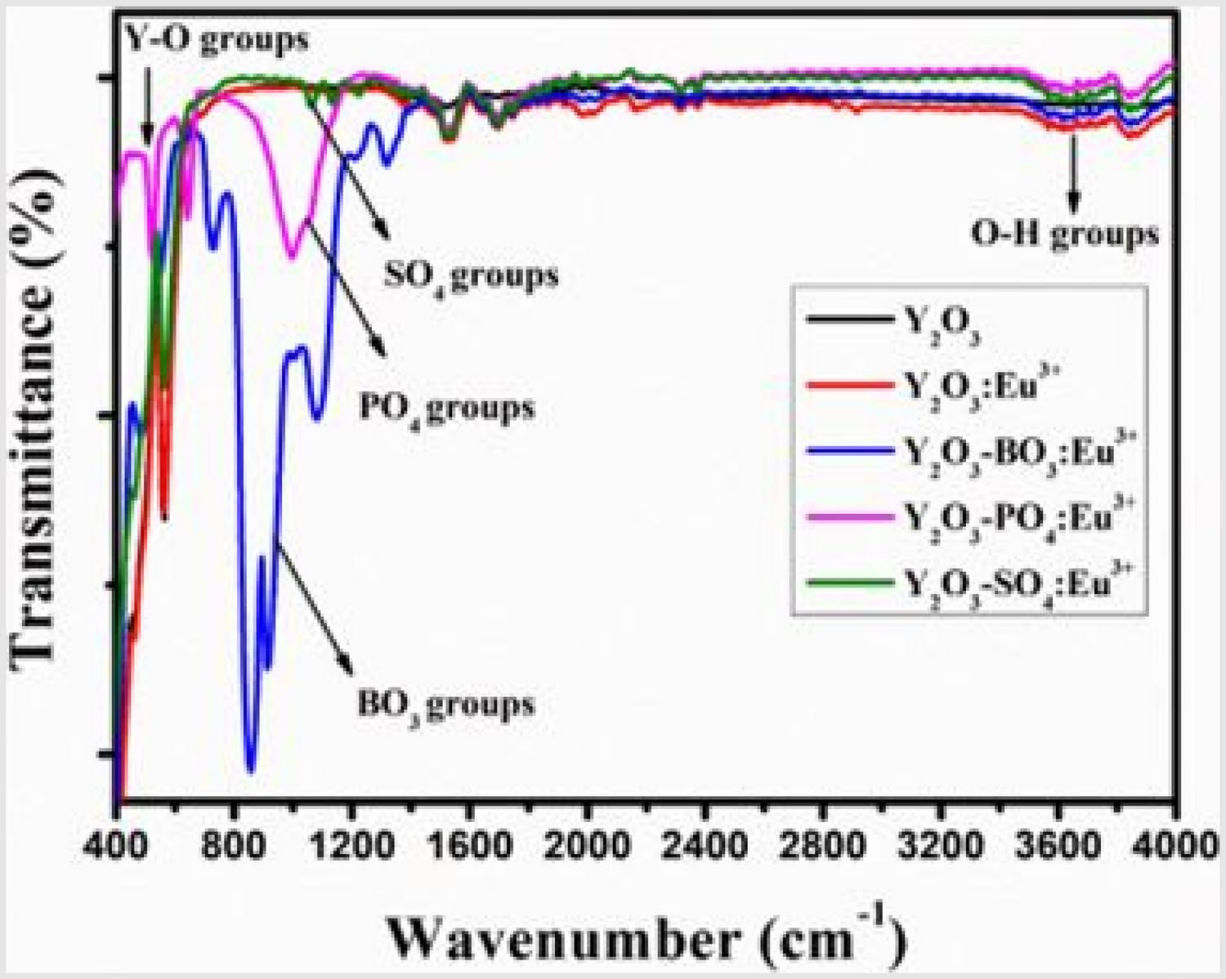
FTIR spectra of the Y_2_O_3_-AG-Eu^3+^ phosphor materials, with AG = (BO_3_^3-^), (PO_4_^3-^), and (SO_4_^2-^) [23] (Reused with permission from [23])

FTIR measurements, as shown in Fig. 19, for the Y_2_O_3_-AG-Eu^3+^ phosphor materials are displayed above. The absorption spectra of Eu^3+^ activated host materials are shown, with Y_2_O_3_-BO_3_^3−^ having the highest absorption, followed by Y_2_O_3_-PO_4_^3−^ and Y_2_O_3_-SO_4_^2−^ groups in that order. This will accordingly affect the photoluminescence properties of the host materials.

**Fig. 20:**
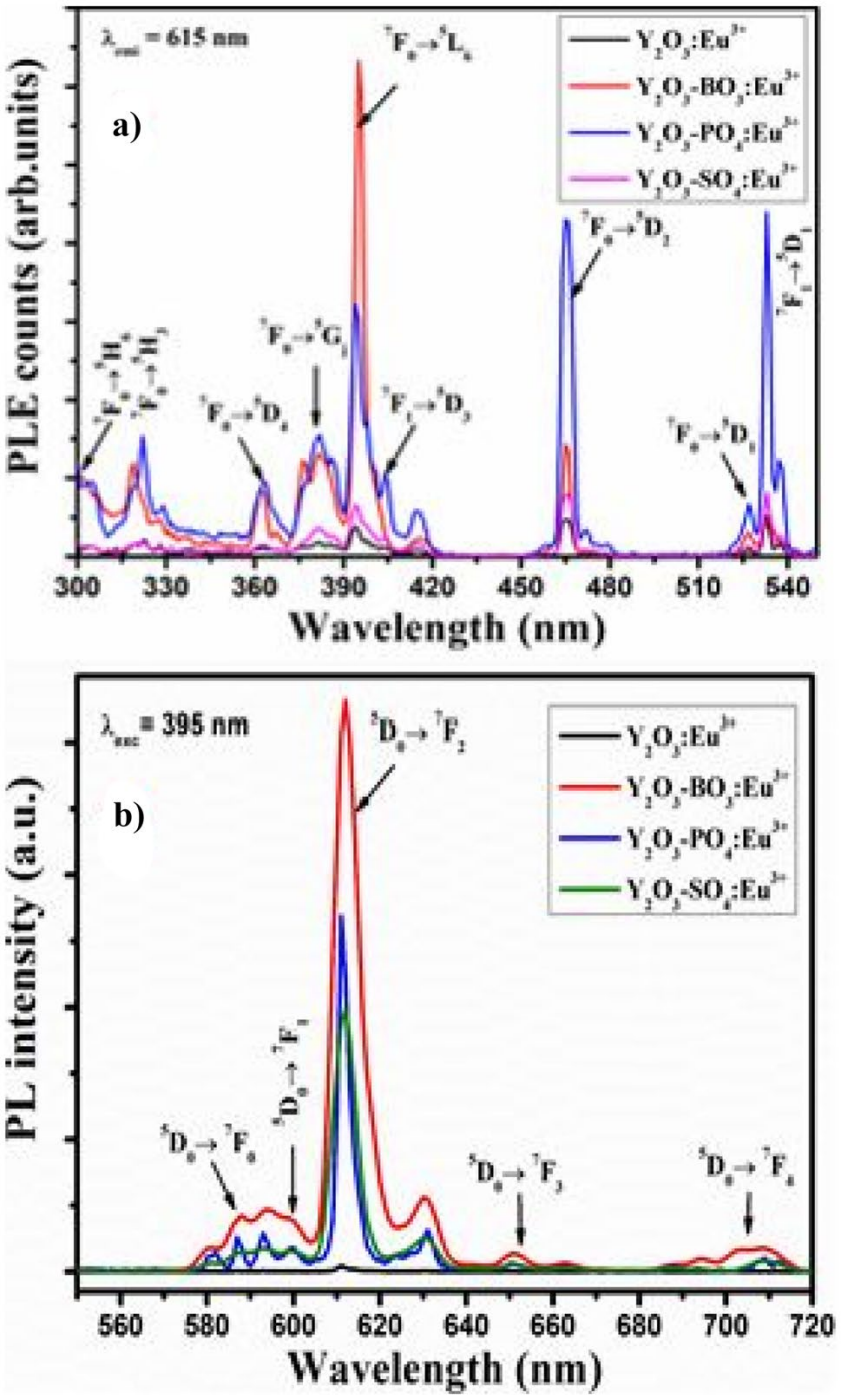
PL excitation and emission spectra of the Y_2_O_3_-AG-Eu^3+^ phosphor materials, with AG =(BO_3_^3-^), (PO_4_^3-^), and (SO_4_^2-^) [23] (Reused with permission from [23])

The PLE excitation and PL emission spectra for the Y_2_O_3_-AG-Eu^3+^ phosphor materials are shown in the diagram below. The excitation band consists of excitation peaks from 300 to 540 nm, which is ascribed to f-f transitions of Eu^3+^ ions in the host material. The most intense excitation peak occurs for the Y_2_O_3_-BO_3_-Eu^3+^ phosphor material at around 395 nm wavelength. Correspondingly, the emission spectra of Eu^3+^ ions consists of emissions bands that occurs in the 560 nm to 720 nm wavelength range. The PL excitation and emission spectra correlates with FTIR measurements and shows that Y_2_O_3_-BO_3_^3−^:Eu^3+^ produces the highest red emission, followed by Y_2_O_3_-PO_4_^3−^-Eu^3+^ and Y_2_O_3_-SO_4_^2−^-Eu^3+^ in transformed crystal structure materials. Borate ions appears to have an advantage over the other anions in producing enhanced luminescence in transformed crystal structure. This means that they have a higher sensitizing effect than the other anions, which makes useful applications in optoelectronic devices. It must be mentioned that borate found in hexagonal structures doped (from cubic structures) with Eu^3+^ ions produces luminescence that is about 100 times better than a single cubic Y_2_O_3_ host material doped with Eu^3+^ ions (see Fig. 20).

### Important Findings of this Study

By comparing the luminescence properties of Y_2_O_3_:Eu^3+^ phosphors doped with different anions, it is observed that Y_2_O_3_-BO_3_^3−^ produces the most favorable photoluminescence characteristics in transformed structures. Thus borates, in this study appears to be a better sensitizer than other anions in capturing excitation energy and transferring it to Eu^3+^ ions non-radiatively and creating a favorable environment for dopant embedding and charge stabilization with the dopants, thus making them better suited for optoelectronic applications. This suggests that borates ions contributes positively to the functionality of these materials, making them ideal for various applications.

## Role of the Single Negative Sulphate Group (SO_4_^2−^) in Sodium Calcium Sulphate Host Na_2_Ca (SO_4_)_2_: Sm^3+^/Eu^3+^ Phosphor Material [24]

In this research, both dopants Sm^3+^ and Eu^3+^ ions are both incorporated into the Na_2_Ca(SO_4_)_2_ phosphor material. Each of these dopants (Sm^3+^ and Eu^3+^) have unique luminescence properties that are characterized by light emission at different wavelengths. By co-doping Na_2_Ca(SO_4_)_2_, our aim was to investigate the interaction between these dopant ions and their effect on the inherent presence of the anions in the host material. The SO_4_^2−^ group is instrumental in facilitating the energy transfer process between the Eu^3+^ and Sm^3+^ dopant ions. Furthermore, the crystal fields created by the SO_4_^2−^ groups affects the electronic transitions of the dopant ions which impacts on its luminescence efficiencies. Its tetrahedral structure further creates stability for dopant embedding.

### Review of the Key Features of this Study

**Fig. 21:**
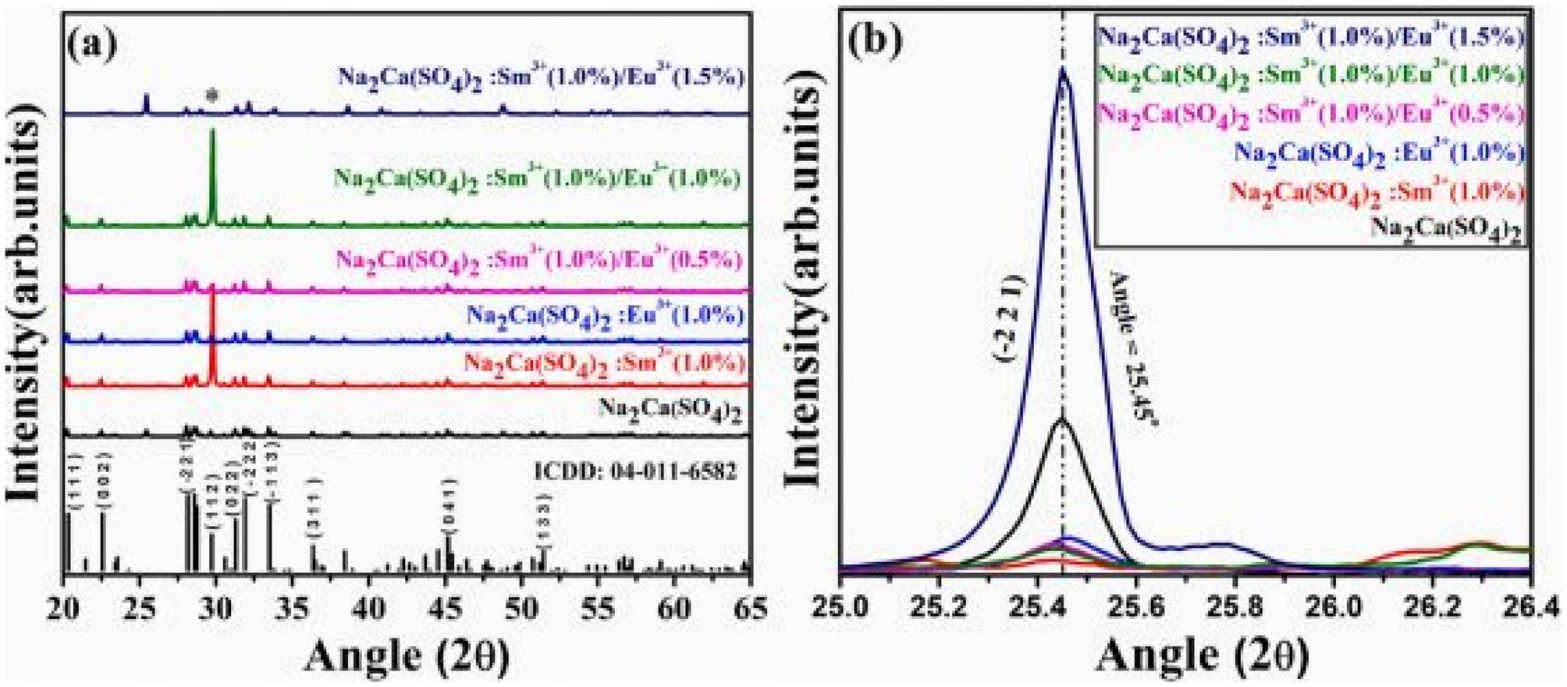
XRD patterns of the Na_2_Ca (SO_4_)_2_: Sm^3+^/Eu^3+^ phosphor material is shown in (**a**), while figure (**b**) is an enlarged section of the (-2 2 1) plane [24] (Reused with permission from [24])

Figure 21(a) shows the XRD patterns of the Na_2_Ca (SO_4_)_2_: Sm^3+^/Eu^3+^ phosphor material, which are crystallized into a monoclinic phase structure, when synthesized by the combustion method. The additions of the dopants (either individually or through a combination) had no impact on the monoclinic phase structure of the host material, thereby conforming its crystalline structures, and dominance in certain planes, a possible anisotropic behavior. However, slight shifts to higher diffraction angles were observed which may be attributable to the differences in the ionic radius of Sm^3+^ (1.08 Å) and/or Eu^3+^ (1.06 Å) replacing the Ca^2+^( 0.99 Å) ions. Impurities were also observed and whose origin may be derived from dopant incorporations. The inherent role of SO_4_^2−^ as a sensitizer is crucial for effective energy transfer between dopants.

The radius of the dopant ion is compared to each of the ions that it is supposed to replace, and so deviation at the lattice site occurs when that substitution has occurred and to see if it is compliant. This is obtained by using the same formula earlier in the section. Thus, by using the formula, the lattice deviations for a coordination number of CN = 6, the ionic radius of the following ions are as follows: Na^+^ (1.02 Å), Ca^2+^ (1.00 Å) and Sm^3+^(0.958 Å). This will give percentage deviations ${\Delta }_{r}$ of 6% & 4.2% for Na^+^ and Ca^2+^ replacements, respectively. If CN = 8, then ${\Delta }_{r}$ would give values of 8.55% and 3.66% for Na^+^ and Ca^2+^ replacements, respectively. These values, either for CN = 6 or 8, which are less than 30% implies that Sm^3+^ could replace either of them (Na^+^ or Ca^2+^) without causing too much crystal distortions.

**Fig. 22:**
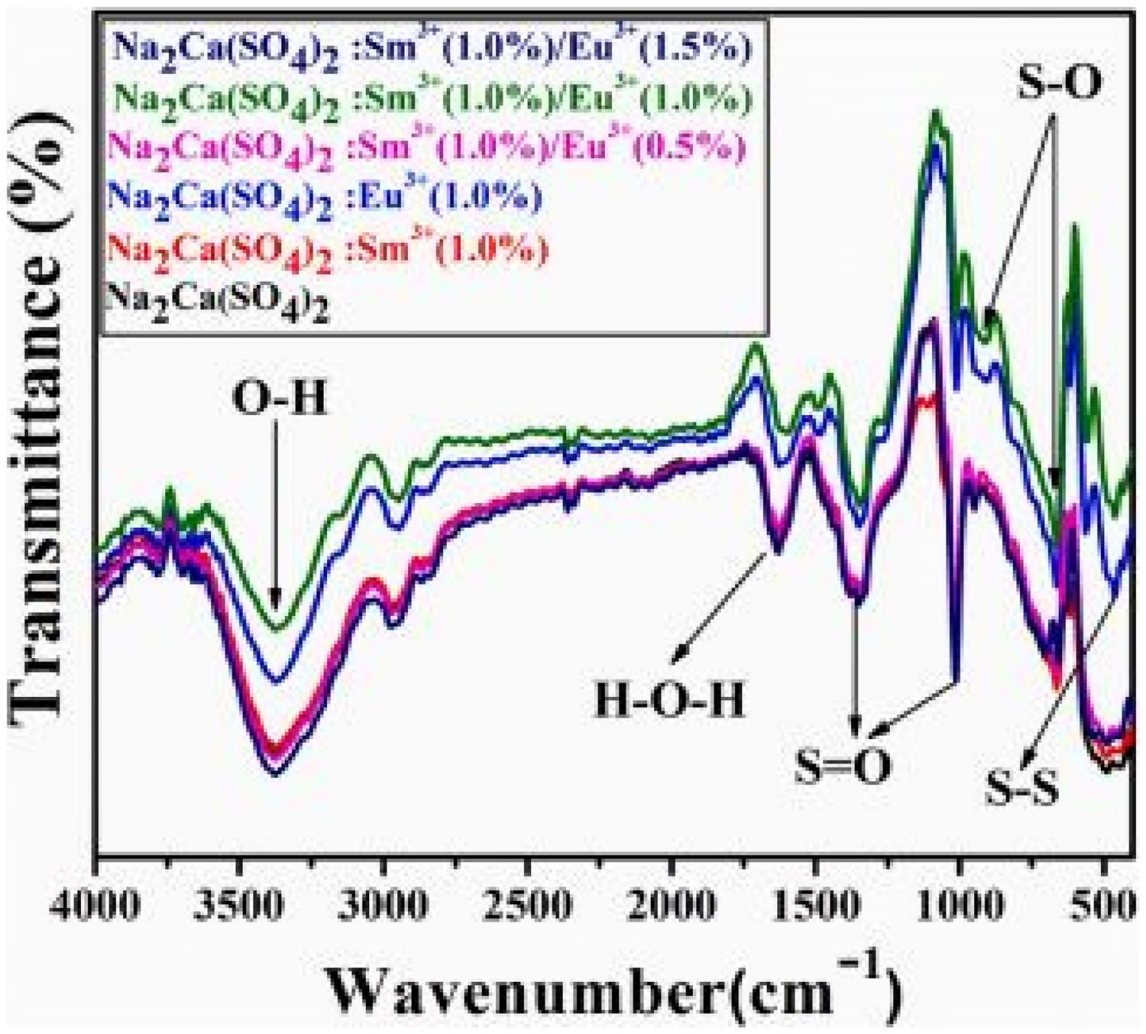
FTIR spectra of the co-doped Na_2_Ca (SO_4_)_2_: Sm^3+^/Eu^3+^ phosphor materials [24] (Reused with permission from [24])

The FTIR measurements of the co-doped Na_2_Ca (SO_4_)_2_: Sm^3+^/Eu^3+^ phosphor material is shown in Fig. 22 below. The figure shows the absorption of the symmetric and asymmetric stretching bands of the sulphate groups, which impacts on its luminescence behavior. The presence of other groups will be responsible for taking the energy away from the luminescence centers and causing quenching effects.

**Fig. 23:**
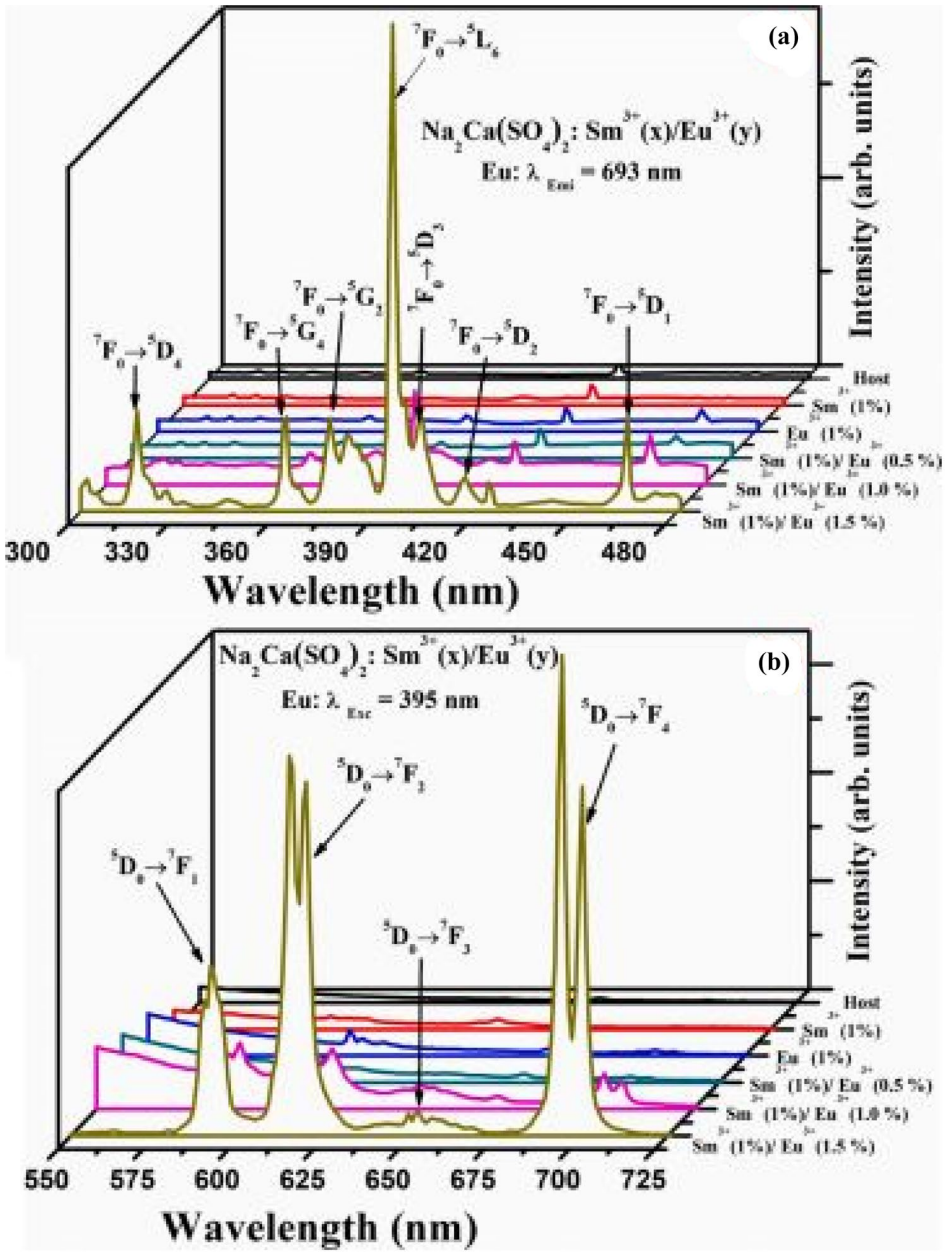
PL excitation and emission spectra of the co-doped Na_2_Ca (SO_4_)_2_: Sm^3+^/Eu^3+^ phosphor materials [25] (Graph taken from reference [25], published in the Journal of Molecular Structure)

The PLE excitation and PL emission spectra of the Na_2_Ca(SO_4_)_2_:Sm^3+^/xEu^3+^ phosphor materials are shown in the figure below. The PLE excitation spectra shows a range of excitation bands, those with Sm^3+^ and Eu^3+^ incorporations, as well as for the co-doped samples. Each unique excitation band reveals f-f transitions either of Sm^3+^(for orange red) or Eu^3+^ (for red) ions transitions in the host material. The most intense excitation band occurs for the co-doped phosphor material, Na_2_Ca(PO_4_)_2_:1 mol%Sm^3+^/1.5 mol%Eu^3+^ at around 400 nm wavelength. As far as the emission spectra is concerned, only the energy transfer between the dopants leads to enhanced red emissions. The impact of the sulphate ions is to produce enhanced red emissions via the spectral overlap between the excitation and emission spectra (around 620-630 nm) of the dopant’s ions. Further, enhanced red emissions are brought about by the efficient energy transfer between Sm^3+^ ions and Eu^3+^ ions. Thus, the role of the anions, as usual, is to act as sensitizer in absorbing the excitation energy and transferring it to the dopants, which is also made possible by the stable environment created by the SO_4_^2−^ anionic group (see Fig. 23).

### Important Findings of this Study

This sensitized effect caused by the SO_4_^2−^ group, combined with the energy transfer mechanism between the Sm^3+^ and Eu^3+^ dopant ions resulted in enhanced red emission properties. Furthermore, this was facilitated by the stable environment created by the SO_4_^2−^ groups for embedding of the dopants. Moreover, SO_4_^2−^ does play a role in limiting the quenching effects, except for the presence of other groups that could take away the energy from the luminescent centers. This red emitting phosphor material finds applications in WLEDS.

## Role of SO_4_^2−^ Ions in Sodium Magnesium Sulphate Host Na_6_Mg (SO_4_)_4_: Doped With Sm^3+^and Eu^3+^ Ions [25]

The role of sulfate (SO_4_^2−^) ions in Na_6_Mg (SO_4_)_4_ is to serve as part of a host lattice for dual doping of Sm^3+^ and Eu^3+^ ions. These Na_6_Mg (SO_4_)_4_ host materials create an environment that is stable for dopant embedding without causing any major structural deformations. Furthermore, the SO_4_^2−^ group also brings about charge stability with dopants with a + 3-charge factor. The stability achieved from the above will allow for efficient energy transfer to take place between the Sm^3+^ and Eu^3+^ dopants, stimulated by the presence of the SO_4_^2−^anion.

### Review of the Key Features of this Study

**Fig. 24:**
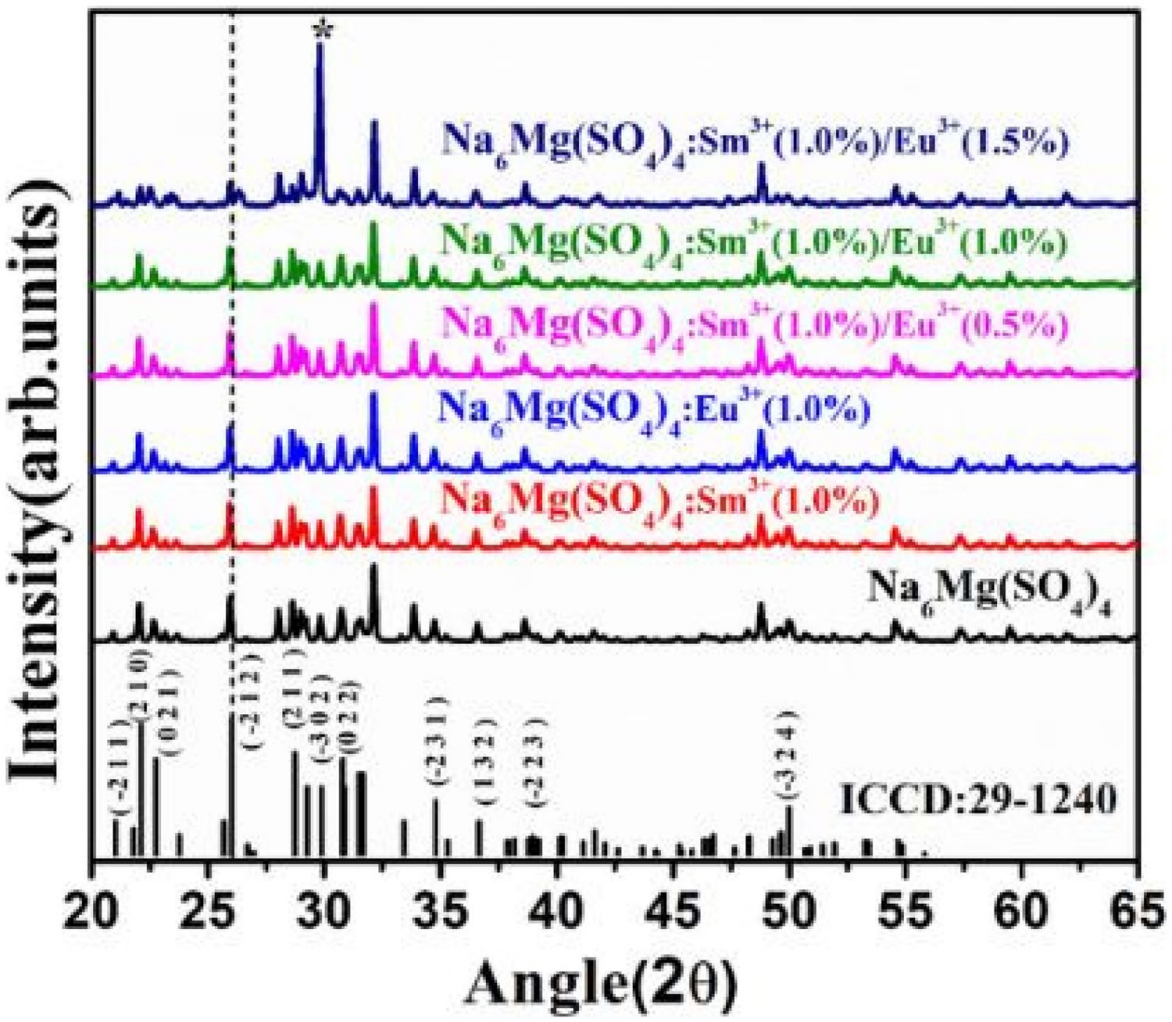
XRD patterns of the Na_6_Mg (SO_4_)_4_: Sm^3+^ phosphor material, co-doped with Eu^3+^ ions [25] (Graph taken from reference [25], published by Inorg. Nano-Metal Chem., 2022)

Figure below show the XRD patterns of the Na_6_Mg(SO_4_)_4_ phosphor material, with separate Eu^3+^ or Sm^3+^ additions or a combination of these dopants. Despite these additions, a monoclinic crystal structure is consistently maintained. No major shifts in the diffraction patterns were observed for either dopant addition, due to the similarity of their atomic radii, even for a combination of these dopants. Observations point towards lowering of some intensity peaks and presence of some impurities due to dopant additions. The inherent role of SO_4_^2−^ as a sensitizer is crucial for effective energy transfer between dopants (see Fig. 24).

The deviation percentage error at lattice sites is obtained using the same formula earlier in the section. For this section, ${\Delta }_{r}$ has a percentage error of 6.07% and 7.15% for CN = 6 when Na^+^ is replaced by Sm^3+^ or Eu^3+^ ions, and a percentage error 33.52% and 19.77% for CN = 8 when Mg^2+^ is replaced by Sm^3+^ or Eu^3+^ ions. Whilst Na^+^ replacements are acceptable, replacements involving Mg^2+^ may cause some slight distortions to the crystal lattice when CN = 8.

**Fig. 25:**
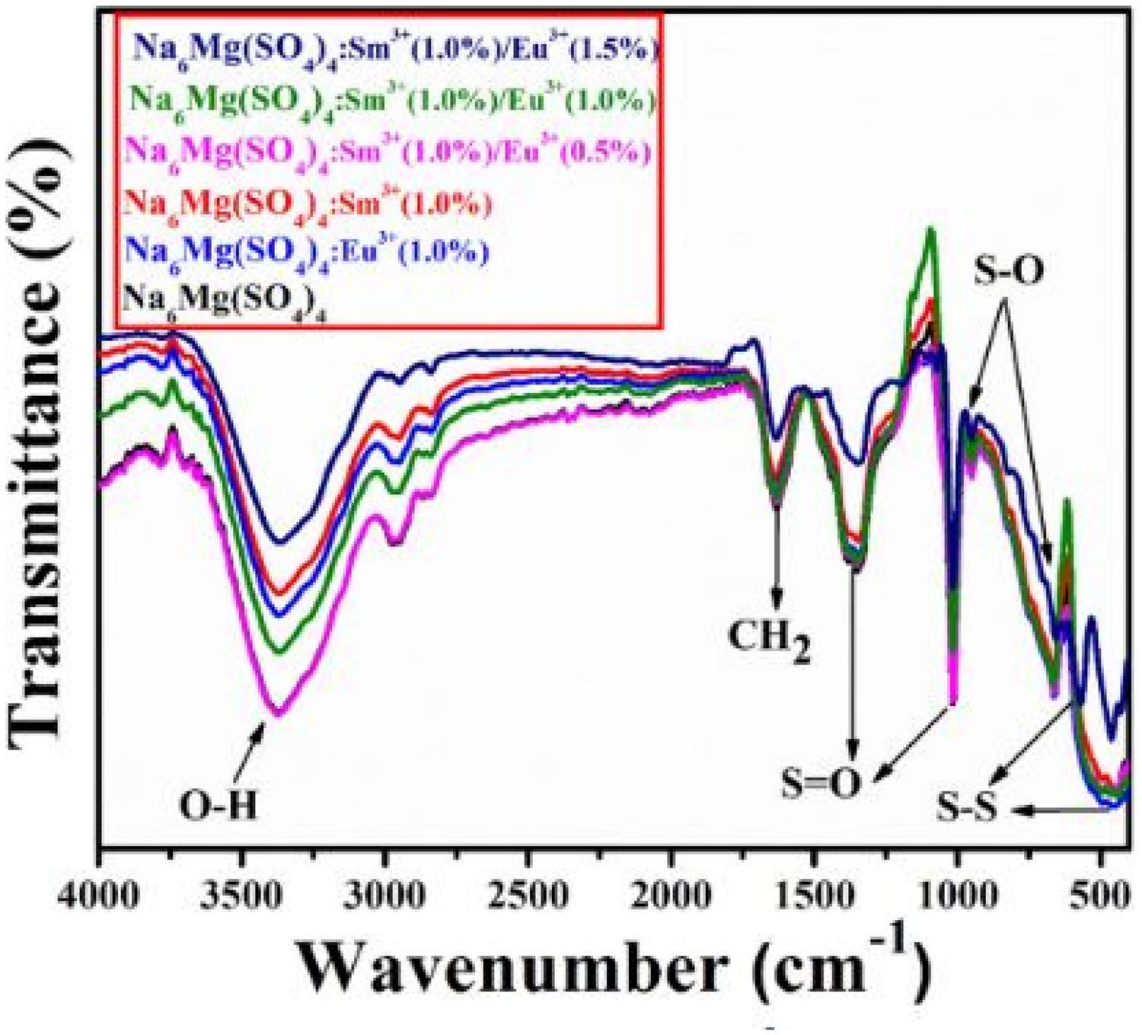
FTIR measurements of the Na_6_Mg (SO_4_)_4_: Sm^3+^ phosphor material, co-doped with Eu^3+^ ions [25] (Graph taken from reference [25], published by Inorg. Nano-Metal Chem., 2022)

Figure 25 shows the FTIR spectra of the Na_6_Mg (SO_4_)_4_: Sm^3+^ phosphor material, co-doped with Eu^3+^ ions. The figure displays symmetric and asymmetric bending vibrations of the S–O groups, as well as the stretching vibrations of the S–O groups. Intense absorptions are displayed by the S–O groups in the host lattice. Furthermore, unnecessary bands account for quenching the PL emissions.

**Fig. 26:**
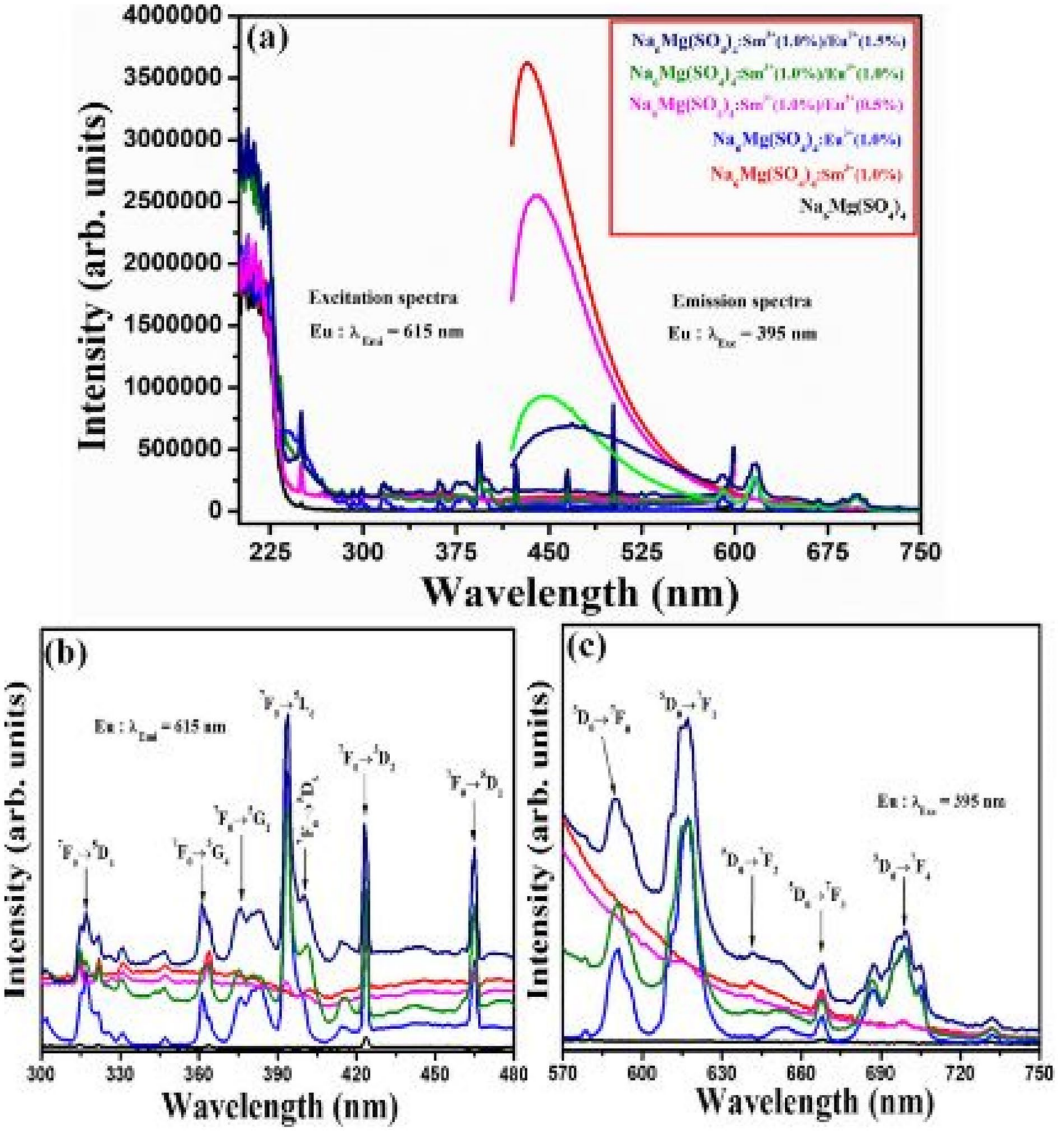
Full PLE and PL spectrum of the Na_6_Mg (SO_4_)_4_: Sm^3+^ phosphor material is shown in (**a**), figure (**b**) shows the PLE spectrum of the phosphor material and **c** shows the PL emission spectra of the material, co-doped with Eu^3+^ ions [25] (Graph taken from reference [25], published by Inorg. Nano-Metal Chem., 2022)

The PLE excitation and PL emission spectra of the Na_6_Mg(SO_4_)_4_ phosphor material is shown in 3 curves; (a) a full spectrum of the PLE excitation and PL emission curves, while (b) and (c) represents enlarged PLE and PL spectra, respectively. We have a very interesting scenario here, as observed in the full-length spectra (a), we observe for the first time the dominance of SO_4_^2−^ emissions over either of the emissions from the dopant ions (Sm^3+^ or Eu^3+^ ion), demonstrating the enhancing character of the SO_4_^2−^ anions. In this spectrum, it is noted that Eu^3+^ ions are excited, while the excitation of Sm^3+^ ions is not shown and is left to the reader to refer to the article, where quite similar trends were observed. In diagram (b), we observe the PLE spectra of various excitation bands due to f-f transition of Eu^3+^ ions in the host material. In diagram (c), we observe that dominant red emission occurring for the co-doped sample (Na_6_Mg(SO_4_)_4_: 1 mol%Sm^3+^/1.5 mol%Eu^3+^) [due to spectral overlap, as shown in the diagram]. The emission intensity is sensitive to crystal field effects in producing red emissions. Enhanced red emissions are produced due to the energy transfer between Sm^3+^ ions to Eu^3+^ ions. The role of sulphate ions is to likewise create a stable environment for the dopants, and its role as sensitizer is crucial in absorbing excitation energy and transferring it non-radiatively to the dopants, but also it produces its own emission (see Fig. 26).

.

### Important Findings of this Study

The role of SO_4_^2−^ in this paper is many-fold. Firstly, it acts as a sensitizer in absorbing excitation energy and transferring it to the Eu^3+^ and Sm^3+^ dopants. Secondly, it creates crystal fields that affects the electronic transition of these dopants. Thirdly, it acts as charge compensator with dopants of charges + 3. Fourthly, it has a tetrahedral structure that allows for stability for dopant embedding. The sensitizing effect of the SO_4_^2−^ group allows for efficient energy transfer to take place between Sm^3+^ ions and Eu^3+^ ions in producing enhanced red luminescence. This red emitting phosphor has potential applications in WLEDs. But more so SO_4_^2−^ is shown to produce its own emissions that supersedes that of the dopant ions.

## Role of Multiple Anions (BO_3_^3−^, PO_4_^3−^, SO_4_^2−^) in CaMo_4_:Sm^3+^ in Doped Phosphor Materials [3]

Each of the anions (BO_3_^3−^, PO_4_^3−^, SO_4_^2−^) interacts with the positive Sm^3+^ ions, which creates crystal fields that modifies the PL emission characteristics of CaMoO_4_:Sm^3+^ phosphor materials by tuning the orange-red emissions due from Sm^3+^ deeper into low intensity red colour emissions. Furthermore, the incorporation of anionic group systems at the atomic site of MoO_4_^2−^ accounts for red colour emission intensity.

### Review of the Key Features of this Study

The XRD results indicate that the CaMoO_4_:AG-Sm^3+^ (AG = BO_3_^3−^, PO_4_^3−^, SO_4_^2−^) phosphor materials are crystallized into a tetragonal structure when synthesized by the solid-state reaction method. There appears to be minimal changes in the diffraction patterns due to small amounts of Sm^3+^ ions and anionic group additions, except for small shifts in the diffraction peaks to higher 2θ values. The reason why there was no changes in the crystal structure was because of two reasons; namely, firstly the radius of Sm^3+^ (0.0964 nm) was similar to Ca^2+^ (0.112 nm) when replaced at atomic sites, and secondly the substitutions of the various anions only caused shift in the diffraction peaks due to strains resulting from different atomic radii; B^3+^ (41 pm) [borate], P^5+^(52 pm) [phosphate] and S^6+^(43 pm) [sulphate] which was much smaller when replacing larger Mo^6+^(73 pm) ions at atomic lattice sites of the host compound. In the whole the following replacements have been affected: each of the anions, BO_3_^3−^, PO_4_^3−^, SO_4_^2−^ replaces the MoO_4_^2−^ ions, while Sm^3+^ ion replaces the Ca^2+^ cationic ions.

**Fig. 27:**
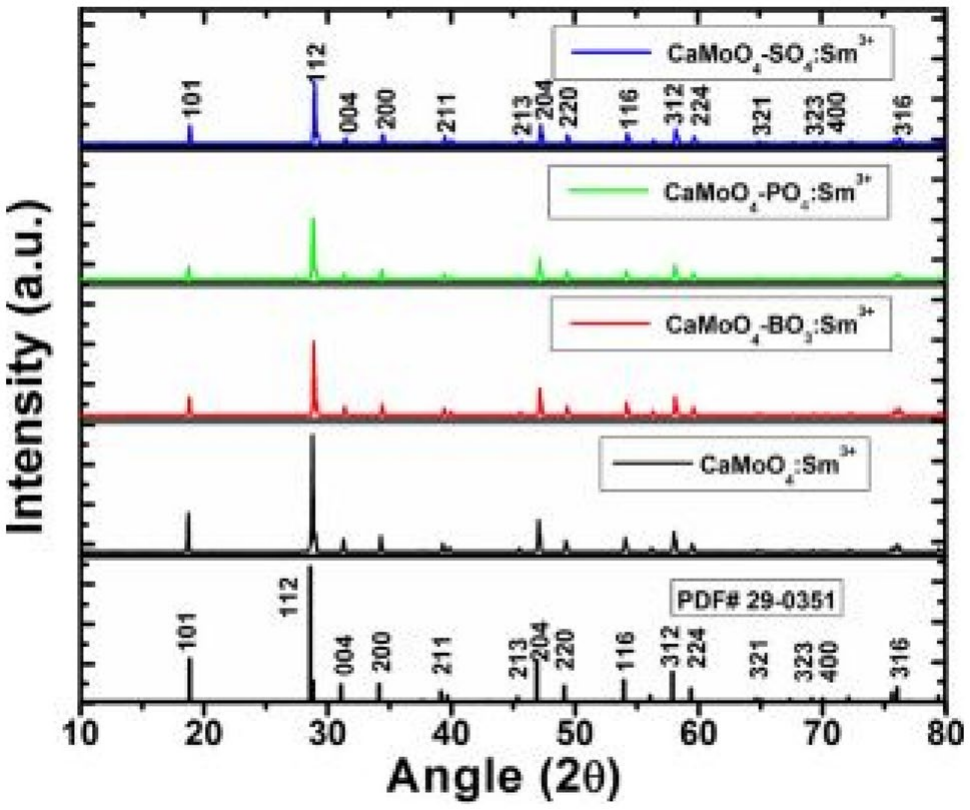
XRD spectra of the CaMoO_4_-AG: Sm^3+^ phosphor materials, with AG = BO_3_^3−^, PO_4_^3−^ and SO_4_^2−^ ions [3] (Reused with permission from [3])

Using an earlier formula, the lattice deviations for a coordination number of CN = 6, with ionic radius of Ca^2+^ (1.00 Å) and Sm^3+^(0.958 Å), gives a lattice deviation of ${\Delta }_{r}$ = 4.20% and a value of 3.66% if CN = 8. These values are very much less than 30% implying good replacements without significant crystal distortions (see Fig. 27).

**Fig. 28:**
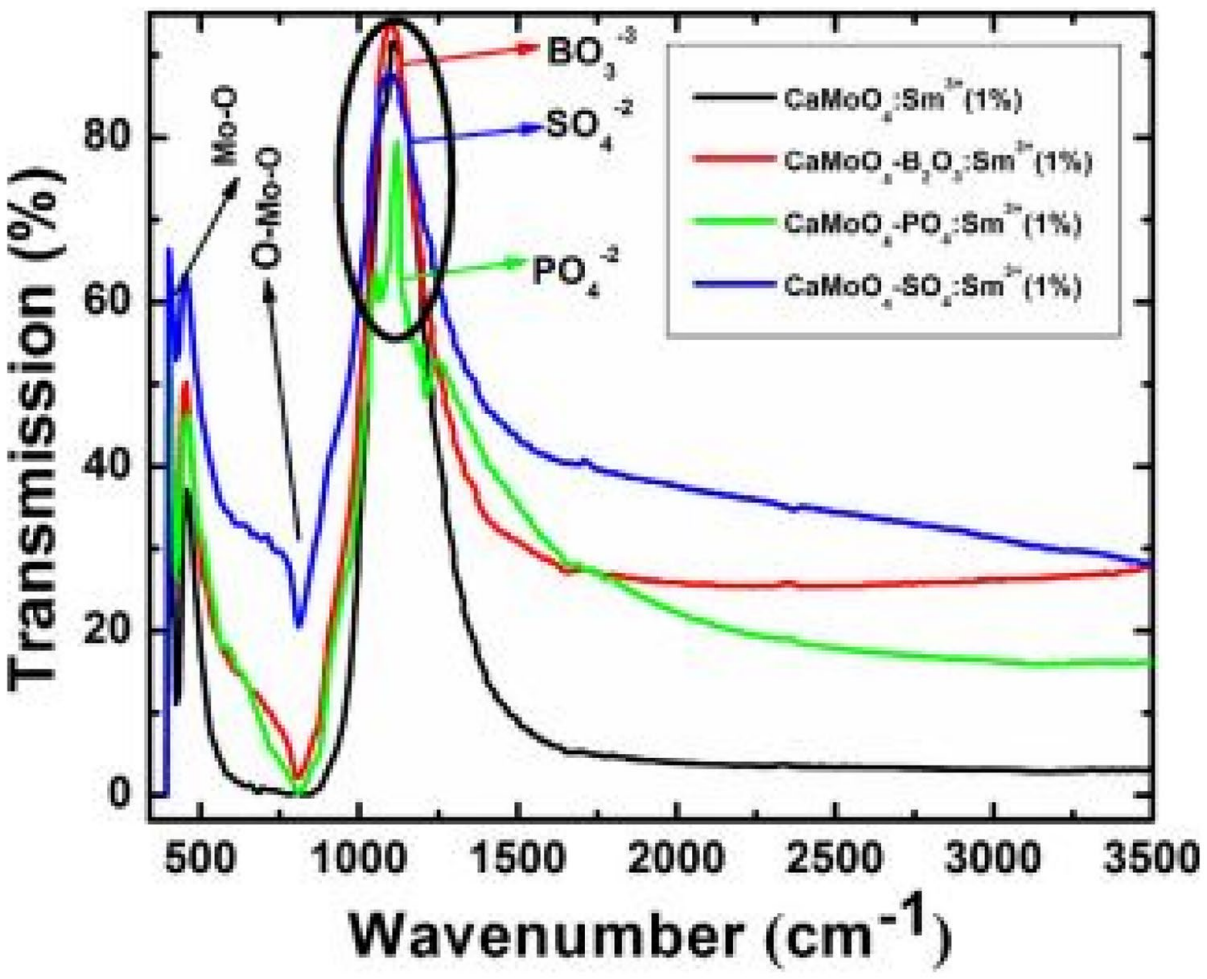
FTIR spectra of CaMoO_4_ -AG: Sm^3+^ (1 mol%) phosphor materials with AG = BO_3_^3-^, PO_4_^3-^ and SO_4_^2-^ ions [3] (Reused with permission from [3])

FTIR measurements show similar absorption peaks for all BO_3_^3−^, PO_4_^3−^, SO_4_^2−^ anions around 1120 cm^−1^. Further, absorption peaks from the host material, located at 424 cm^−1^ and 811 cm^−1^, are attributed to the Mo–O and O–Mo-O stretching vibrations of these respective functional groups. The positioning of all anionic group absorption is due to a good replacement of MoO_4_^2−^ groups for each of the respective anions in crystal matrix (see Fig. 28).

**Fig. 29:**
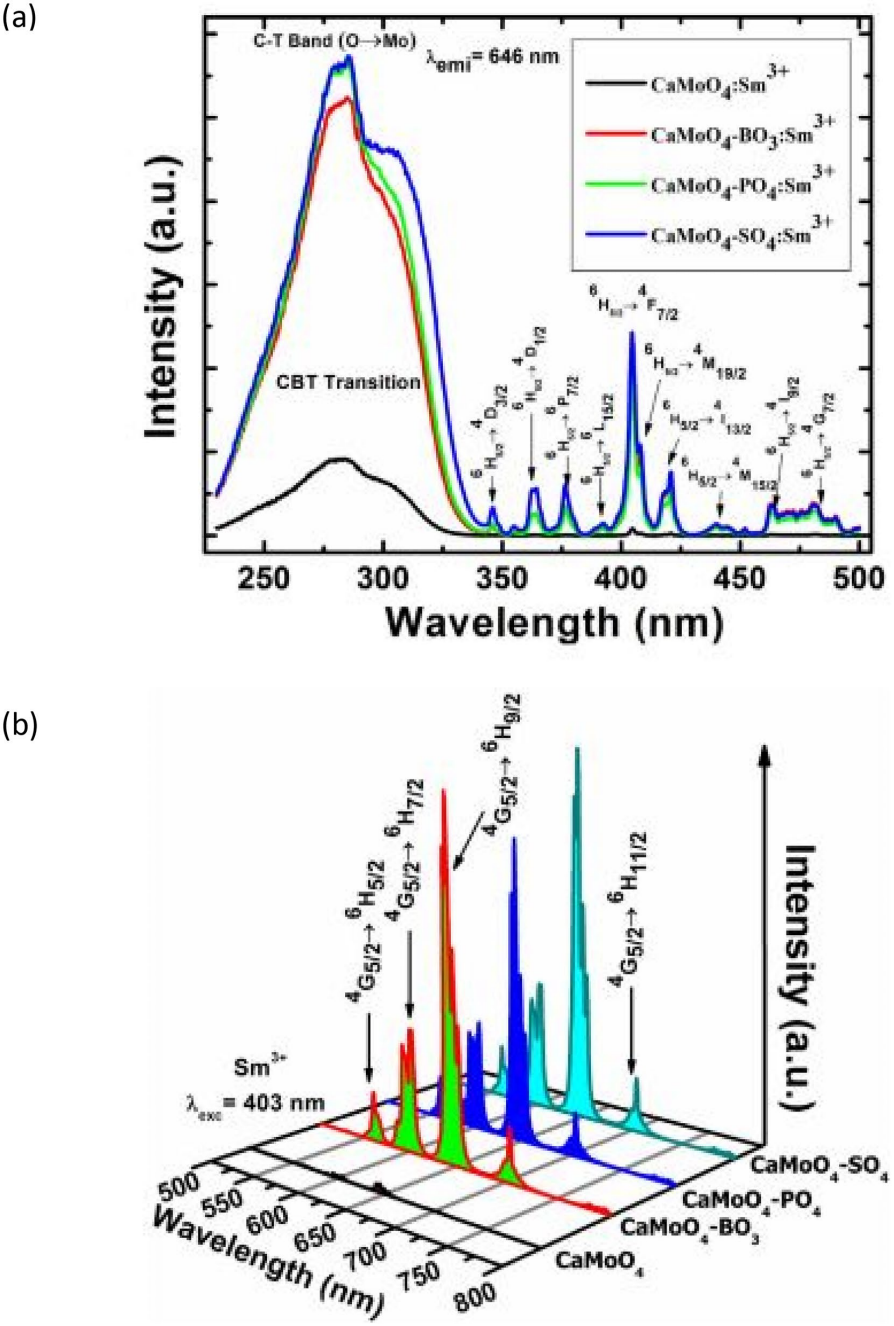
Figure **a** shows the PLE excitation spectra and figure **b** shows the PL emissions spectra of the CaMoO_4_ -AG: Sm^3+^ (1 mol%) phosphor materials with AG = BO_3_^3-^, PO_4_^3-^ and SO_4_^2-^ ions [3] (Reused with permission from [3])

The figure for the PL measurements shows both the excitation and emission spectra of the CaMoO_4_:AG-Sm^3+^ (AG = BO_3_^3−^, PO_4_^3−^, SO_4_^2−^) phosphor materials. The PLE excitation spectra shows a distinct peak at 286 nm, which is attributed to the charge transfer band (CTB), arising from O^2−^ and Sm^3+^ and O^2−^ and Mo^6+^ recombination, while other varying excitation bands are due to Sm^3+^ transitions from the ground state to various higher excited states. On the other hand, the PL emission spectra show a range of (f-f) emissions from the metastable state of Sm^3+^ to its ground state. Whilst the interaction of Sm^3+^ with the host, is supposed to create crystal fields that affects the electronic transitions, but observing their peak and shape of these curves implies that such as not occurred, hence anionic additions did not influence the electronic transitions but has an enhancing effect. As such the enhancement is about 30 times greater than for an un-anionic doped CaMoO_4_:Sm^3+^ host materials (see Fig. 29).

### Important Finding of this Research

Anionic substitutions did not influence the tetragonal crystal structure of the host CaMoO_4_: Sm^3+^ phosphor material, but modified its optical properties to remarkable levels, when synthesized by the solid-state reaction method. Notably the following substitutions have transpired for these phosphor materials: Sm^3+^ → Ca^2+^ (lack of charge compensation) and SO_4_^2−^/BO_3_^3−^/PO_4_^3−^ → MoO_4_^2−^. The PL intensity for red emissions was 30-fold higher than for un-anionic substitutions and with improved quantum efficiency. Of all anionic substitutions, the CaMoO_4_:SO_4_-Sm^3+^ phosphors produced the best red emissions, making them ideally suitable for LED applications and is comparable to the commercial InGaN chip used to produce white LEDs.

## Role of Anions (PO_4_^3−^) and (SO_4_^2−^) as Mutually Varying Parameters in Ca_3-x_Li_x_(PO_4_)_2-x_(SO_4_)_x_:Dy^3+^, Sm^3+^ Phosphor Materials [14]

The role of both the phosphate and sulphate ions in this stoichiometric formula is interesting as one quenches the other. The role of Dy^3+^ doping is to produce white light when appropriate ratios of blue (at 487 nm) and yellow (587 nm) light are combined, but the role of Sm^3+^ is tune the light away from white light towards orange-red regions for higher concentrations of Sm^3+^ ion. The nature of the white light produced from Dy^3+^ will depend on the optimized concentrations of both these anions, with Sm^3+^ ions absent.

### Review of the Key Features of the Study

The XRD patterns show that the samples are crystallized into an orthorhombic structure when synthesized by the sol–gel method. All data are consistent with the standard background Ca_3_(PO_4_)_2_ crystal structure. However, weak impurities due to Ca_5_(PO_4_)_3_(OH) were observed. The addition of charge compensators such as Li^+^ and S^6+^ or Dy^3+^ lead to no additional peaks as they were of small concentrations. When the concentration of x raged from 0 to 0.2 mol%, there was a shift to larger diffraction angles (smaller volume), implying crystal distortion. This was attributed to them having a similar ionic radius of Dy^3+^ (0.091 nm), Ca^2+^ (0.099 nm), Li^+^ (0.076 nm), P^5+^ (0.038 nm) [phosphate] and S^6+^ (0.037 nm) [sulphate] ions. The replacement of Li^+^/S^6+^ (combined 0.113 nm) for larger Ca^2+^/P^5+^ (combined 0.137 nm) ions has caused lattice contraction and shifts to higher diffraction peaks. Thus, the additions of Li^+^ and S^6+^ ions has led to higher diffraction intensities and improved crystallinity. No FTIR studies were done for these samples.

**Fig. 30:**
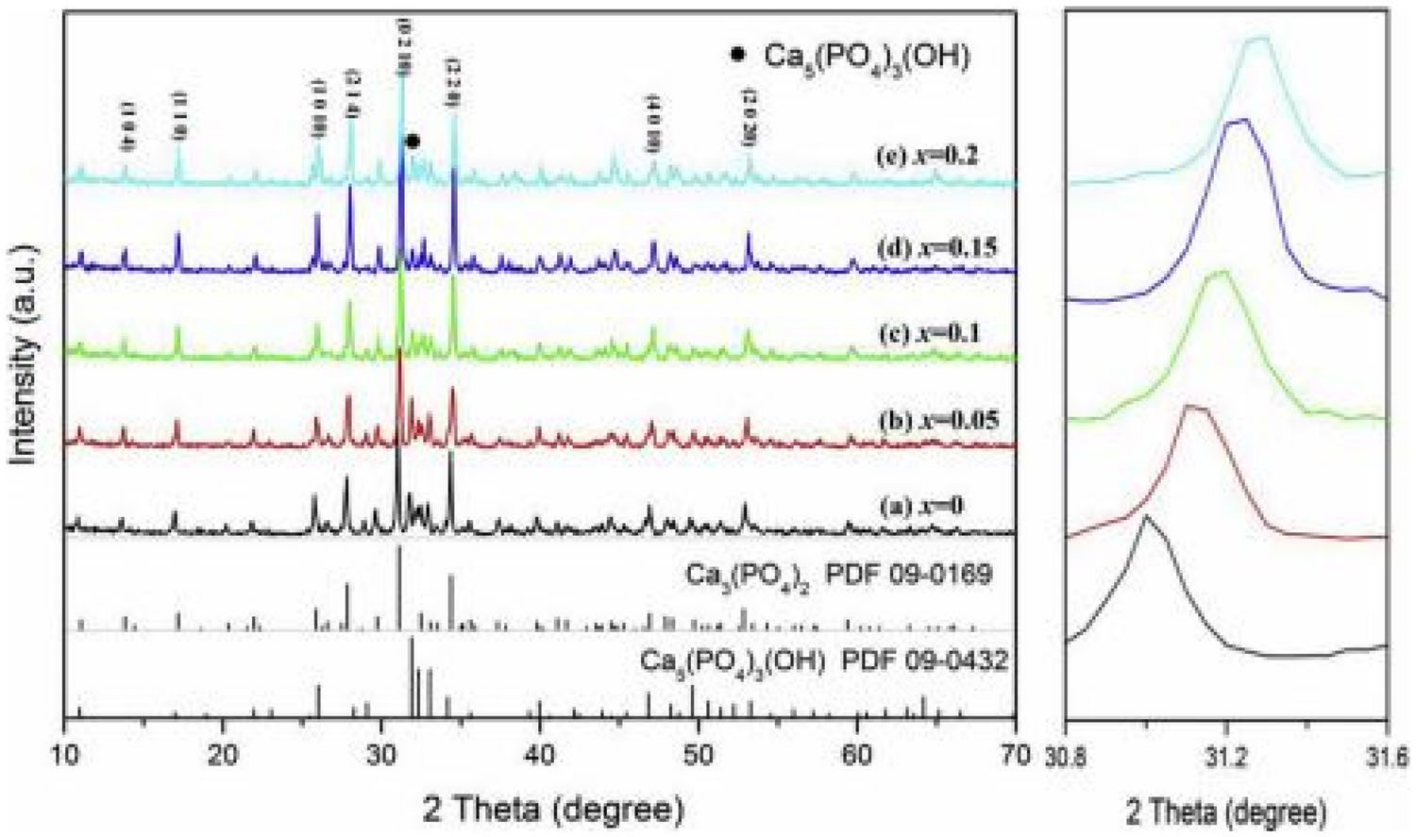
XRD spectra of the Ca_3-x_Li_x_(PO_4_)_2-x_(SO_4_)_x_:Dy^3+^, Sm^3+^ phosphor materials [14] (Reused with permission from [14])

The deviation percentage at lattice sites is obtained using the same formula earlier in the section. For this section, ${\Delta }_{r}$ has a percentage error of 8.80% and 4.20% for CN = 6 when Ca^2+^ is replaced by Sm^3+^ or Dy^3+^ ions and a percentage error 14.46% and 8.30% for CN = 8 when Ca^2+^ ions is replaced by Sm^3+^ or Dy^3+^ ions. These results are acceptable, implying that either Sm^3+^ or Dy^3+^ could replace Ca^2+^ ions in the crystal lattice without distortions (see Fig. 30).

**Fig. 31:**
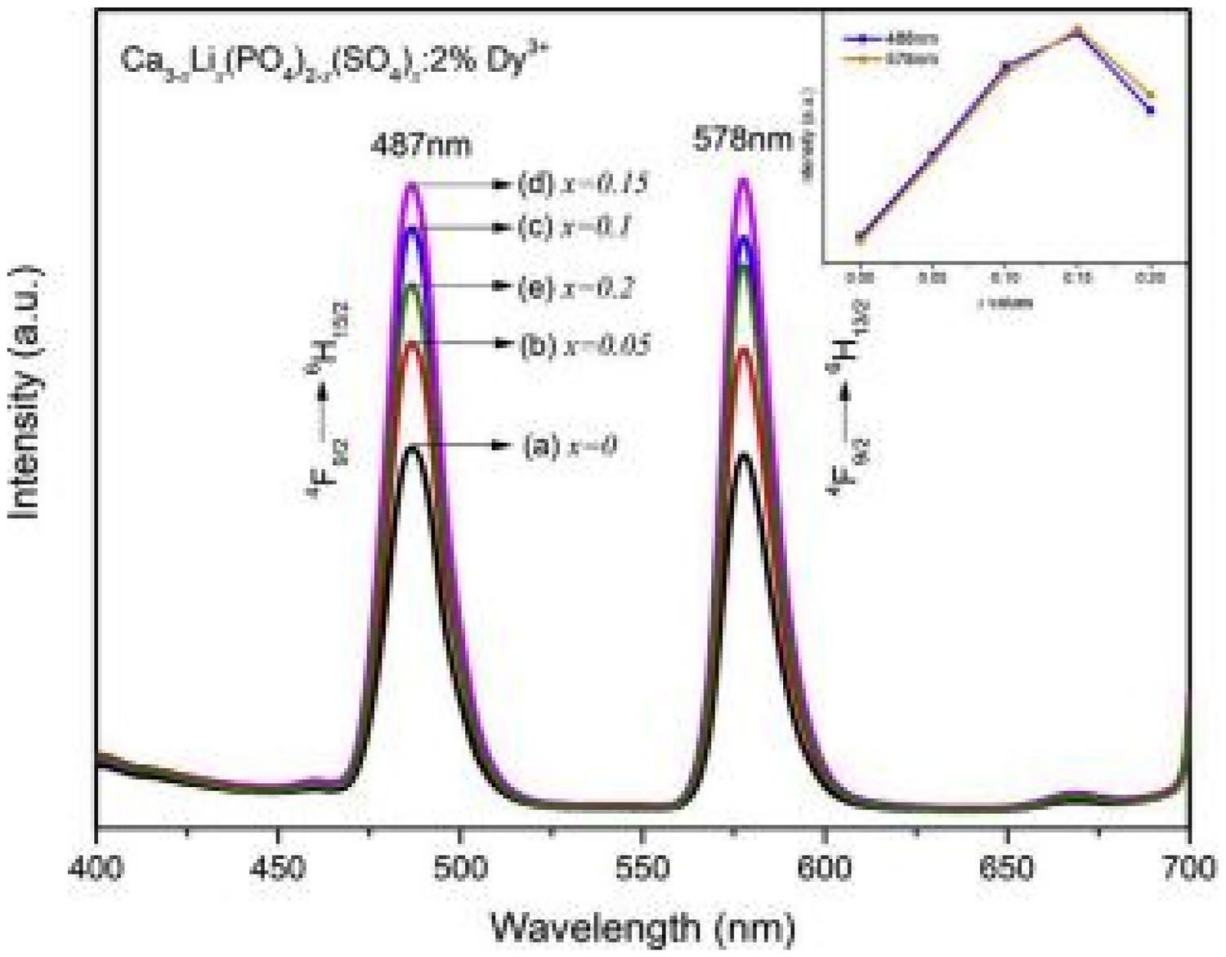
PLE excitation and PL emission spectra of the Ca_3-x_Li_x_(PO_4_)_2-x_(SO_4_)_x_:Dy^3+^, Sm^3+^ phosphor materials [14] (Reused with permission from [14])

The PL emission spectra for these samples are both shown in the graph. The emission spectra shows that as x is increased from x = 0 to 0.15 mol%, a blue maximum emission peak is observed at 487 nm and a further maximum yellow emission peak at 578 nm, both occurring for a maximum concentration of x = 0.15 mol% SO_4_^2−^ ions and 1.85 mol% PO_4_^3−^ ions. In this scenario, as the concentration of the SO_4_^2−^ anions increased, the concentration of the PO_4_^3−^ ions decreased, which all occurs for concentration of 2 mol%Dy^3+^ ions. Thus, the impact of Li^+^ and S^6+^ ions brought about some lattice distortions, which causes changes in the electronic fields surrounding the Dy^3+^ ions which has resulted in enhanced white colour emissions (see Fig. 31).

### Important Finding of this Study

All prepared materials were synthesized by the sol–gel method and matched well with the standard background structure of Ca_3_(PO_4_)_2_. Further, the additions of Dy^3+^ or Sm^3+^ has not altered the crystal structure. The replacement of Li^+^/P^5+^[phosphate] by Ca^2+^/S^6+^ [sulphate] ions has significantly improved the luminescence properties of these materials for concentration up to a maximum of x = 0.15 mol% [SO_4_^2−^, x = 1.85 mol% PO_4_^3−^]. For concentrations higher than this value leads to concentration quenching, whereby the impurity Ca_5_(PO_4_)_3_(OH) will play a more significant role in producing non-radiative transitions and a decrease in luminescence.

## Role of Heterovalent [PO_4_]^3−^ → [SO_4_]^2−^ Anionic Substitutions in β-Ca_3_(PO_4_)_2_ Type of Phosphor Materials [26]

In this work mixed sulphates and phosphates of stoichiometric formulas, Ca_10.5–0.5x_(PO_4_)_7-x_(SO_4_)_x_ and Ca_9.5–0.5x_Mg(PO_4_)_7-x_(SO_4_)_x_ have been synthesized by the solid-state reaction method (with x = 0, 0.1 and 1). To study the influence of SO_4_^2−^ ions on the luminescence properties, additional series with stoichiometries of Ca_9_Me(PO_4_)_6_(SO_4_):5%Eu^3+^, and Ca_9.5_Me(PO_4_)_7_:5%Eu^3+^ (Me = Mg^2+^, Zn^2+^) were synthesized. In the latter, the roles of the cations in bringing about charge stabilization is considered.

### Review of the Key Features of this Study

**Fig. 32:**
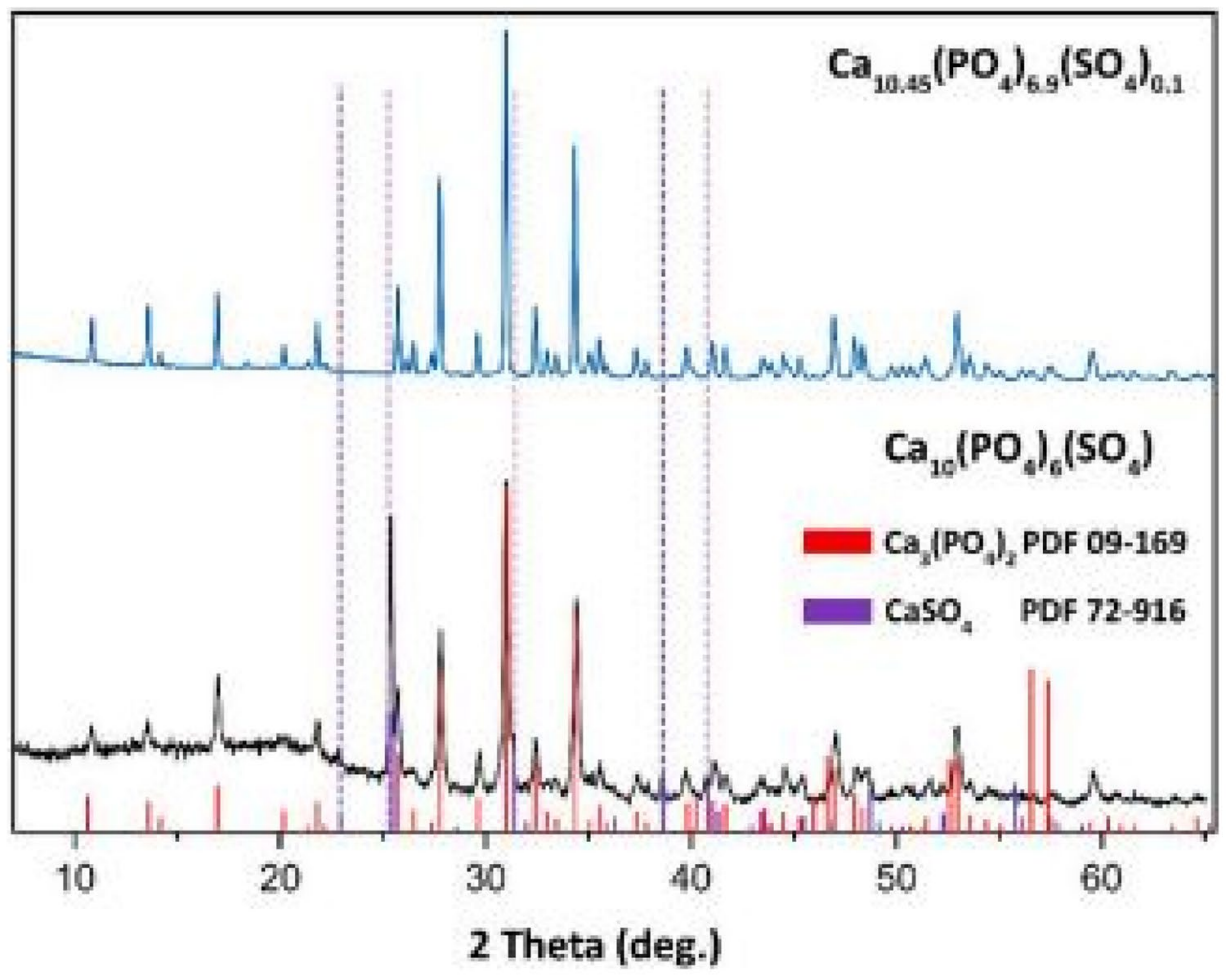
XRD spectra of the Ca_10.5–0.5x_(PO_4_)_7-x_(SO_4_)_x_ and Ca_9.5–0.5x_Mg(PO_4_)_7-x_(SO_4_)_x_ phosphor material for x = 1 mol% [26] (Reused with permission from [26])

It is observed that a significant amount of CaSO_4_ impurity was formed in the Ca_10_(PO_4_)_6_(SO_4_) materials, however, the additions of SO_4_^2−^ negates such formation. This is attributed to the ionic radius of S^6+^ (0.12 Å) [sulphate] being smaller than P^5+^ (0.15 Å) [phosphate] (SO_4_^2−^ ion replacing PO_4_^3−^ ion). In addition to this, the synthesis of the other phosphors with smaller cationic radius (than Ca^2+^), such as Me = Mg^2+^ and Zn^2+^ will help in bringing about structural stability [27, 28]. Further, the additions of these cations also negates the formation of the 2 impurities, namely, CaSO_4_ and MgSO_4_ (see Fig. 32).

The deviation percentage at lattice sites is obtained using the same formula earlier in the section. For this section, ${\Delta }_{r}$ has a percentage error of 5.30% for CN = 6 and a percentage error 4.82% for CN = 8 when Ca^2+^ is replaced by Eu^3+^ ions. These replacements are acceptable without many distortions to the crystal lattice.

**Fig. 33:**
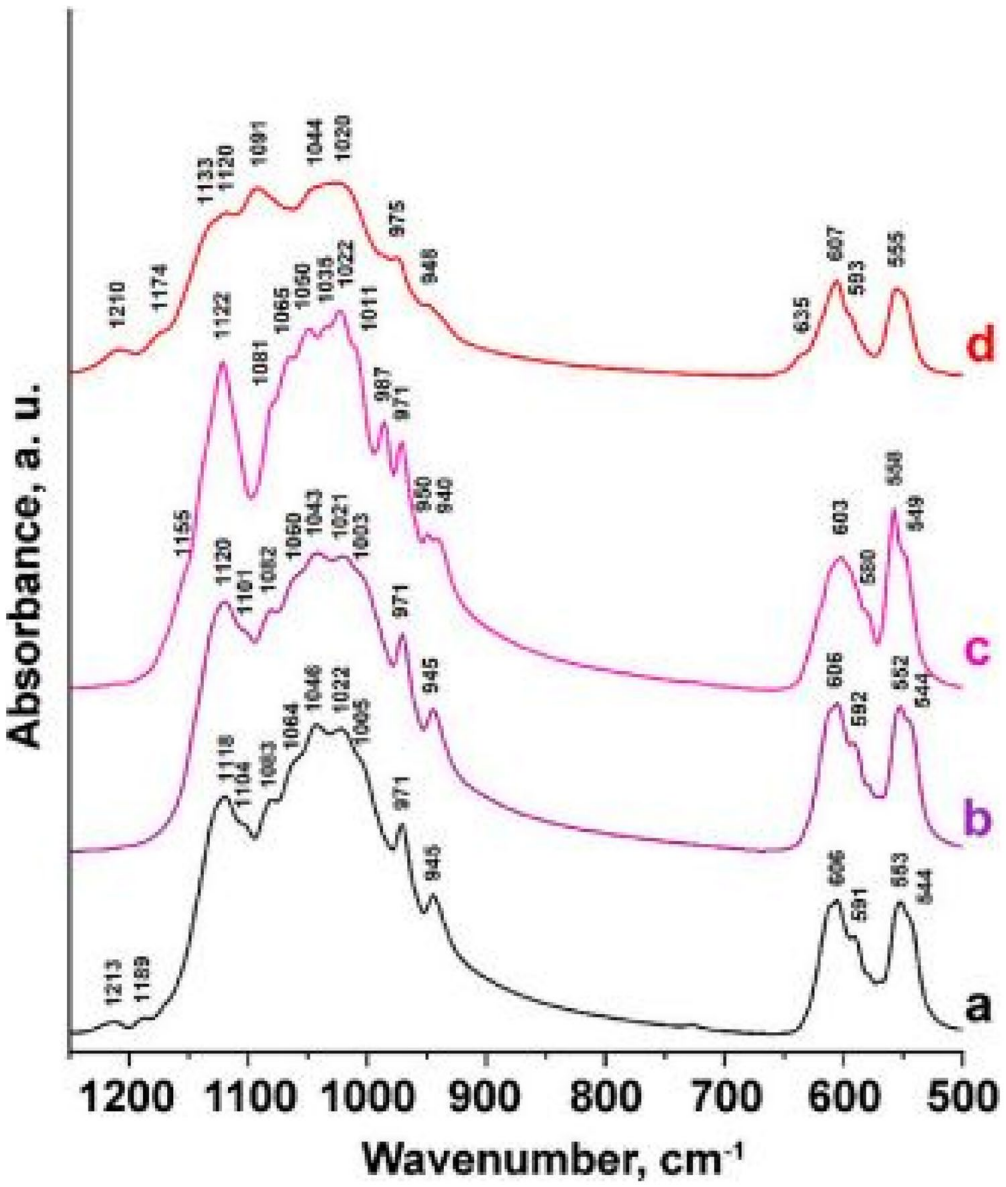
FTIR spectra of the Ca_10.5-0.5x_(PO_4_)_7-x_(SO_4_)_x_and Ca_9.5-0.5x_Mg(PO_4_)_7-x_(SO_4_)_x_ phosphor material for x = 1 mol% [26] (Reused with permission from [26])

FTIR measurements, shows the spectra of (a) β-Ca_3_(PO_4_)_2_, (b) Ca_10.45_(PO_4_)_6.9_(SO_4_)_0.1_, (c) Ca_9.45_ Mg(PO_4_)_6.9_(SO_4_)_0.1_ and (d) Ca_9_Mg(PO_4_)_6_(SO_4_). This study was done to confirm the presence of SO_4_^2−^ groups in the crystal structure. The vibration frequencies associated with SO_4_^2−^ vibrations (S–O at 938, 450, 1105, 611 cm^−1^) are slightly higher than vibration frequencies associate with the PO_4_^3−^ group (P-O at 938, 420, 1018, 567 cm^−1^). This may be due to the differences in the charges between P^5+^ and S^6+^ ions, when these atoms are located at different coordination sites in the tetragonal matrix. This means that the additions of the anions (PO_4_^3−^, SO_4_^2−^) has caused distortions of the crystal lattice, which is significantly observed in figure (d), where a widespread absorption band is observed for the addition of SO_4_^2−^ tetrahedral ion group. Evidence of SO_4_^2−^ anions is also observed within the 635 cm^−1^ spectral region (see Fig. 33)

**Fig. 34:**
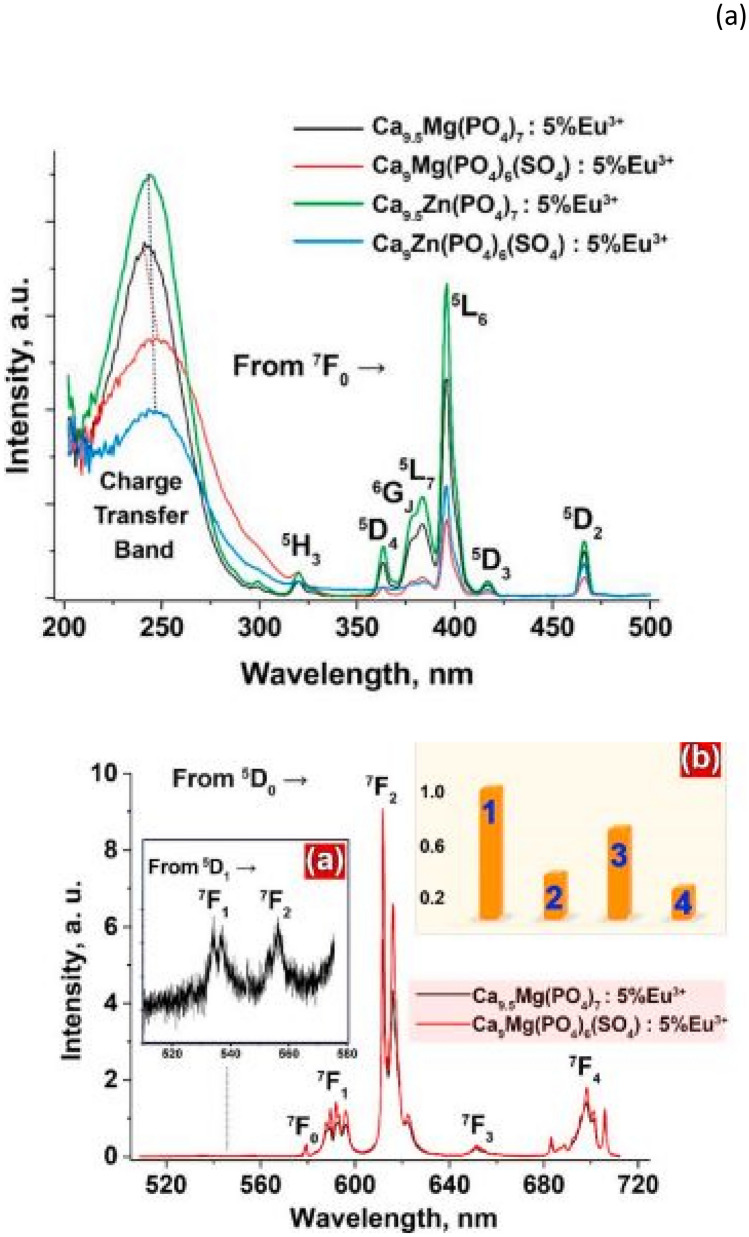
PLE excitations spectra of the prepared material is shown in (**a**), while the PL emission spectra is shown in figure (**b**) [26] (Reused with permission from [26])

The PLE excitation and PL emission spectra of the prepared materials are shown alongside. The PLE spectra shows a comparison of excitations for Ca_9_Me(PO_4_)_6_(SO_4_):5%Eu^3+^ and Ca_9.5_Me(PO_4_)_7_:5%Eu^3+^, (Me = Mg^2+^, Zn^2+^) materials. The PLE excitation spectra consists of a charge transfer band (200–290 nm) and f-f transitions associated with Eu^3+^ ions (290–470 nm) from its ground state. The addition of SO_4_^2−^ caused 2 things, namely, firstly a shift to higher wavelength values, and secondly, it causes reductions in the luminescent intensities, up to 65% for both series with Zn^2+^ and Mg^2+^ ions. The most intense emission was caused with Zn^2+^ cationic inclusion compared to Mg^2+^ inclusion. The role of SO_4_^2−^ in this case was to suppress the luminescence, but the main reason for that was due to polyhedral distortions caused by SO_4_^2−^ inclusions (see Fig. 34).

### Important findings of this study

The role of anionic SO_4_^2−^ substitutions is interesting for β-Ca_3_(PO_4_)_2_- type of crystal structures. The additions of SO_4_^2−^ was found to decrease the luminescent intensities of these type of structures. In particular, the incorporations of smaller cations, such as Mg^2+^ and Zn^2+^ has had a more remarkable effect on the luminescence properties than anionic additions. The addition of Zn^2+^ compared to Mg^2+^ has exhibited an intense in emission intensity by 1.5-fold times in comparison with Mg^2+^ additions. This may be attributed to the presence of charge compensators, such as Li^+^ and the differences between the rare earth ions radii (Eu^3+^ and Dy^3+^).

### The Role of Anions from These 2 Research Papers [25 and 26] on the Luminescent Properties of the Phosphor Materials

It can be generally summarized as follows:

1. It has been shown that *anionic tetrahedral substitutions* ([XO_4_]^n−^) has made a significant contribution to luminescence of compounds such as apatite and β-Ca_3_(PO_4_)_2_ types of structures. Here, such substitutions allows for preservation of the crystal structures, when heterovalent [PO_4_]^3−^ ion is substituted for [SO_4_]^2−^ ions, despite its different in oxidation states (but similar *covalency*). Whilst this may be true, it must be compensated by cationic additions to preserve its charge neutrality ([PO_4_]^3−^ + Ca^2+^ = [SO_4_]^2−^ + M^+^) [26].
2. From [26] it is known that [SO_4_]^2−^ additions was found to increase the luminescence intensities through replacement of S^6+^ [SO_4_]^2−^ charge with P^5+^ [PO_4_]^3−^ charges [29]. This mixed sulphate and phosphate anionic inclusion is significant for its luminescent improvements, due to distortions it creates around the luminescent centers for apatite type structures [26].
3. This result is important for mixed occupancy of tetrahedral sites by sulphates and phosphates and their interactions with each other, that produces improved luminescent properties.
4. Whilst in the previous paper, the replacement of P^5+^ with S^6+^ ions had a positive effect on luminescence, in this instance we see the role of cations (Zn^2+^ and Mg^2+^ that could replace Ca^2+^ ions) have a more dominant effect.

## The Role of the Anion SO_4_^2−^ in a Non-calcium Based Eu^3+^ Activated LiY(PO_3_)_4_ Phosphor Materials [13]

In this paper Eu^3+^ doped LiY(PO_3_)_4(1-x)_SO_4x_ is prepared by the solid-state reaction method. This paper is reviewed to give us a sense of what happens when anions are substituted in non-calcium-based phosphor materials. We observe some similarity in behaviour of how this compound behaves when the concentrations of the anions [SO_4_]^2−^ increases for phosphate [PO_3_]^2−^ replacement.

### Review of the Key Features of this Study

The XRD results of this prepared material, prepared by the solid-state reaction methods, reveals a monoclinic crystal structure. The additions of Eu^3+^ (1.066 Å) for Y^3+^ (1.019 Å) ions and SO_4_^2−^ for PO_3_^2−^ shows no changes in the diffraction patterns due to similarity in the ionic radius of the ions. However, the addition of SO_4_^2−^ leads to crystal structure re-ordering, which seem to have an impact in improving its structural rigidity and thermal stability. Furthermore, once the concentration of SO_4_^2−^ exceeds its optimal value of x = 0.35 mol%, other impurity crystal phases appear and causes luminescence quenching. No FTIR measurements were done for this paper.

**Fig. 35:**
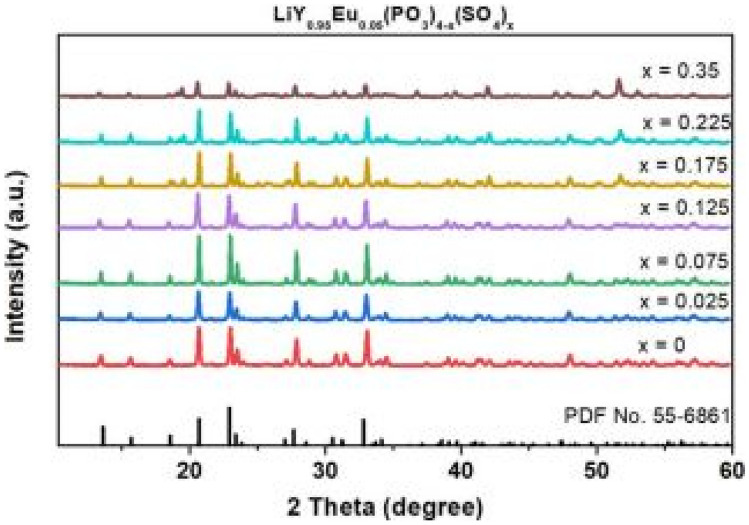
XRD spectra of the Eu^3+^ activated LiY(PO_3_)_4(1-x)_SO_4x_ phosphor materials for varying concentrations of SO_4_^2-^ ions [13] (Graph taken from reference [13], published by ECS J Solid State Sci. Technol., 2023)

The deviation percentage at lattice sites is obtained using the same formula earlier in the section. For this section, ${\Delta }_{r}$ has a percentage error of 24.61% and 4.96% for CN = 6 when Li^+^ and Y^3+^ ions are replaced by Eu^3+^ ions and a percentage error 15.86% and 4.61% for CN = 8 when Li^+^ and Y^3+^ ions are replaced by Eu^3+^ ions. These replacements are acceptable without causing much crystal distortion. No FTIR measurements were done for this sample (see Fig. 35).

**Fig. 36:**
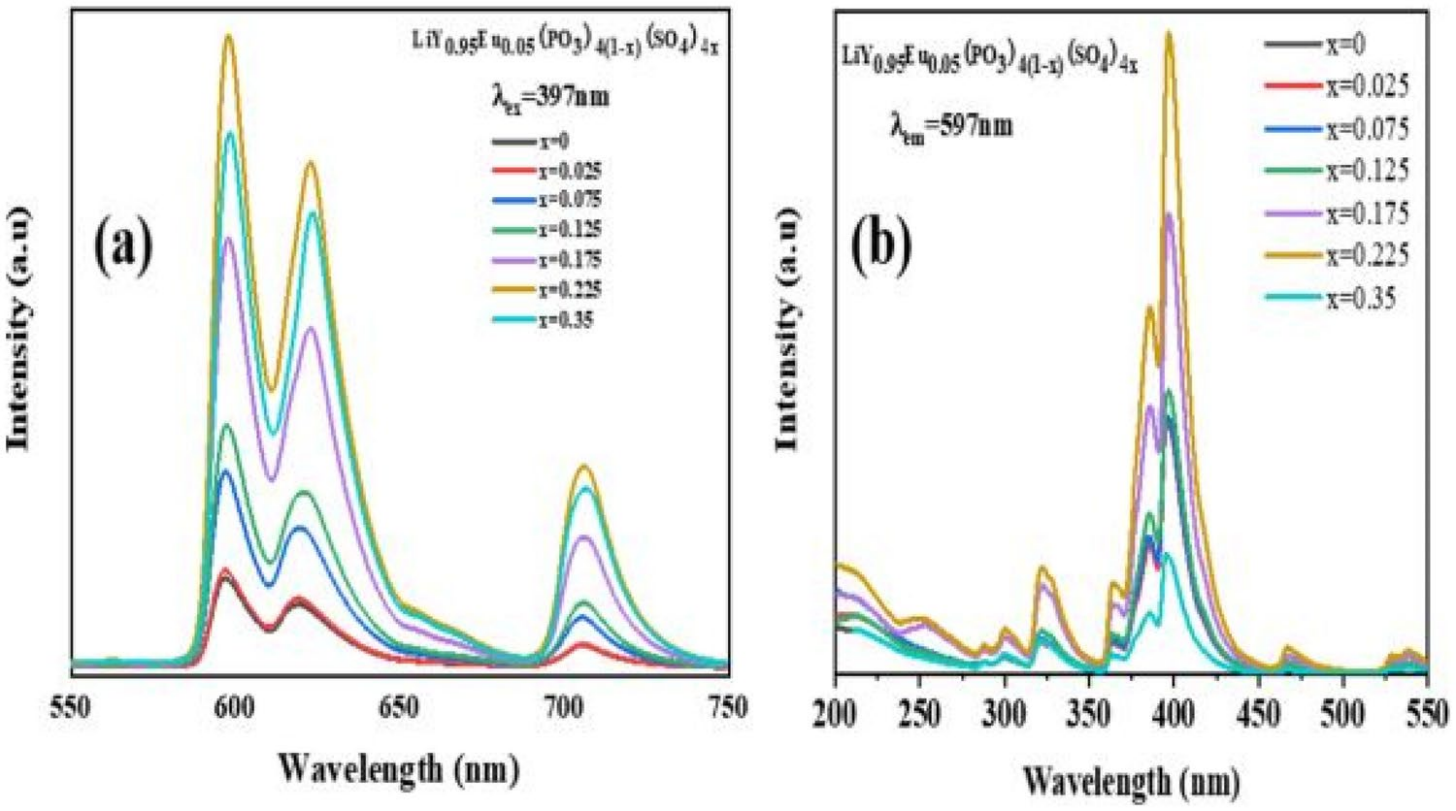
PLE (first) and PL spectra (second) of the Eu^3+^ activated LiY(PO_3_)_4(1-x)_SO_4x_ phosphor materials for varying concentrations of SO_4_^2-^ ions [13] (Graphs taken from reference [13], published by ECS J Solid State Sci. Technol., 2023)

The PLE excitation and PL emission spectra of the prepared are shown alongside. The PLE spectrum shows the usual charge transfer band from 200 to 280 nm, and f-f transitions from 300 to 550 nm which are attributed to f-f transitions of Eu^3+^ ions from its ground state to higher states. The PL emission band shows prominent red emissions at around 610 nm due to Eu^3+^ ions. As the concentrations of [SO_4_]^2−^ ions increases for [PO_3_]^2−^ replacements, there is gradual increase in the luminescent intensities up to an optimized concentration of x = 0.225 mol%. The continuous increase of Eu^3+^ and SO_4_^2−^, the crystal site of Eu^3+^ is not affected, implying that the anionic additions did not affect the electronic transitions of the host material, but enhanced its luminescent properties. The impact of this addition is to increase the intensity of the undoped sulphate material by 7.58 times. It is further stated that anionic [SO_4_]^2−^ doping has led to improved rigidity of the crystal structure, shrinking of the unit cell, thereby promoting a crystal structure that allows for more absorptions to take place for the purpose of enhancing its luminescence characteristics (see Fig. 36).

### Important Findings of this Study

The introduction of [SO_4_]^2−^ into the host matrix through concentration variations, has enhanced its luminescence characteristic by 7.58 times compared to other phosphor materials without sulphates for an optimal concentration of x = 0.225 mol%. The impact of such introductions is to induce crystal distortions, tuning the rigidity of the host matrix, and promoting a compact crystal structure without structural changes for enhancing it luminescence properties and reducing the effects of thermal quenching. For higher concentrations of [SO_4_]^2−^ beyond the optimal concentration of 0.225 mol%, more anions creates more distortion and with more traps, the impact of which is an increase in non-radiative transitions that reduces the luminescence intensities of the phosphor material.

## Conclusion

In conclusion, the incorporation of anions, such as BO_3_^3−^, PO_4_^3−^ and SO_4_^2−^ either individually or as an integral component of the host matrix, has had a significant impact on its luminescence properties. For dopant activated hosts, these anions appear to play a sensitizing role in absorbing energy and transferring it non-radiatively to the dopants, thereby enhancing its luminescent features. The incorporation of dopants such as Ce^3+^, Sm^3+^, and Eu^3+^ for their red-emitting properties finds useful applications in advanced WLED technology. The cations, when incorporated into various hosts, play a fundamental role in altering the crystal structure of the host material, thereby influencing its luminescent features. Overall, anions have played a significant role in all the materials studied, enhancing luminescent properties through their sensitizing effects, charge compensation effects, crystal field effects, and the diverse crystal structures they create.

## Data Availability

N/A.

## References

[1] Xiao F, Xue YN, Ma YY, Zhang QY (2010) Ba_2_Ca(B_3_O_6_)_2_:Eu^2+^, Mn^2+^: A potential tunable blue-white-red phosphor for white light-emitting diodes. Phys B 405(3):891–895

[2] Hu SH, Lin YS, Su SH, Dai H, He JS (2022) Study on the enhancement of light intensity and high color rendering index of a white light emitting diode. J Electron Mater 52:270–275

[3] Balakrishna A, Swart HC, Ramaraghavulu R, Bedyal AK, Kroon RE, Ntwaeaborwa OM (2017) Structural evolution induced by substitution of designated molybdate sites (MoO_4_^2-^) with different anionic groups (BO_3_^3-^, PO_4_^3-^ and SO_4_^2-^) in CaMoO4:Sm3+ phosphors – A study on color tunable luminescent properties. J Alloy Compd 727:224–237

[4] Caisheng X, Shikao S, Huili G, Ji Z (2010) Preparation and luminescent properties of Na_5_Eu(MoO_4_)_4–x_(PO_4_)_x_ red phosphors for white-emitting diodes application. Mater Sci Forum 654–656:2025–2028

[5] Yan Z, Shikao S, Jing G, Ji Z (2010) Effects of SO_4_^2-^ or SiO_3_^2-^ doping on the photoluminescence of NaEu(MoO_4_)_2_ nanophosphors for light-emitting diodes. J Nanosci Nanotechnol 10(3):2156–3160 20355646 10.1166/jnn.2010.2106

[6] Light Emitting Diodes (LED) – Working, construction and symbol-Diode, retrieved 13 Nov 2023. [https://www.physics-and-radioelectronics.com/electronicsdiodes/lightemittingdiodeledconstructionworking.html]

[7] Cho J, Park JH, Kim JK, Schubert EF (2017) White light-emitting diodes: History, progress, and future. Lase Photonics Rev 11(2):1600147

[8] M Wood, How do white LEDs work? Out of the Wood, 2011. [https://wwwmikewoodconsulting.com/articles/Protocol%20Winter%202011%20-%20White%20LEDs.pdf]

[9] Light-emitting diode, retrieved Wikipedia 13 Nov 2023. [https://en.wikipedia.org/wiki/Light-emitting_diode]

[10] Bender VC, Marchesan TB, Alonso JM (2015) Solid-State Lighting, A concise Review of the State of the Art on LED and OLED modelling, retrieved 13 Nov 2023

[11] Zhang M, Chen Y, He G (2014) Color Temperature Tunable White-Light LED Cluster with Extrahigh Color Rendering Index. Hindawi Publishing Corporation, The Scientific World Journal 10.1155/2014/897960 PMC3919051 24578665

[12] White Light Emitting Diode or White LED light, Electrical 4U, retrieved 13 Nov 2023. [ https://www.electrical4U.com/white-led/]

[13] Qiano X, Zhao J, Xu C, Liu Y, Tsuboi T (2023) Emission Enhancement of Eu^3+^ Ions in LiY(PO_3_)_4_ phosphate Phosphors via Anionic SO_4_^2-^ Substitutions. ECS J Solid State Sci Technol 12:066010

[14] Yu M, Xu X, Zhang W, Chen X, Zhang P, Huang Y (2020) The effect of Sm^3+^ co-doping on the luminescence properties of Ca_2.85_Li_0.15_(PO_4_)_1.85_(SO_4_)_0.15_:Dy^3+^ white-emitting phosphors. J Alloys Compd 817:152701

[15] Balakrishna A, Reddy L, Ntwaeaborwa OM, Swart HC (2020) Remarkable influence of alkaline earth ions on the enhancement of fluorescence from Eu^3+^ ion doped in sodium ortho-phosphate phosphors. J Mol Struct 1203

[16] Blasse G (1968) Energy transfer in oxidic phosphors. Phys Lett A 28(6):444–445

[17] Letswalo MLA, Reddy L, Balakrishna A, Swart HC, Ntwaeaborwa OM (2021) Influence of SO_4_^2-^ anionic group substitution on the enhanced photoluminescence behaviour of red emitting CaMoO_4_:Eu^3+^ phosphor 854:157022

[18] Zhang X, Cao C, Zhang C, Xie S, Xu G, Zhang J, Wang X-J (2010) Photoluminescence, and energy storage traps in CaTiO_3_:Pr^3+^. Mater Res Bull 45:1832–1836

[19] Maleka PM, Reddy L, Nkosi TJ, Balakrishna A, Kroon RE, Swart HC, Ntwaeaborwa OM (2020) Structural and morphological characterization of photoluminescent cerium-doped near UV-blue sodium orthophosphate phosphors. J Lumin 226:117409

[20] Reddy L, Nkosi TJ, Masiteng PL, Balakrishna A, Swart HC, Ntwaeaborwa OM (2020) Violet-blue-shift of emission and enhanced luminescent properties of Ca_3_(PO_4_)_2_:Ce^3+^ phosphor induced by substitution of Gd^3+^ ions. Curr Appl Phys 20(5):696–702

[21] Letswalo MLA, Reddy L, Balakrishna A, Swart HC, Ntwaeaborwa OM (2021) The role of sulfate ions on distinctive defect emissions in ZnO:Ce^3+^ nanophosphors – A study on the application on color display systems. J Lumin 240:118462

[22] Letswalo MLA, Reddy L, Balakrishna A, Swart HC, Ntwaeaborwa OM (2019) Effect of BO_3_^3-^ ion on photoluminescence of CaMoO_4_:Eu^3+^ phosphor. J Vac Sci Technol 37(1):012907

[23] Mathe TG, Balakrishna A, Mamo MA, Ntwaeaborwa OM, Kroon RE, Coetzee E, Swart HC, Reddy L (2024) A study on the impact of [BO_3_^3-^], [PO_4_^3-^] and [SO_4_^2-^] ions in normal cubic structures via structural and photoluminescence properties of Y_2_O_3_:Eu^3+^ phosphors. J Mol Struct 1299:137127

[24] Morebodi KB, Reddy L, Balakrishna A, Erasmus LJB, Swart HC, Masiteng PL (2022) Investigation of photoluminescence properties and energy transfer in Sm3+ and Eu^3+^ co-doped Na_2_Ca(SO_4_)_2_ nanophosphors. Solid State Sci 134:167056

[25] Morebodi KB, Reddy L, Letswalo MLA, Balakrishna A, Erasmus LJB, Swart HC, Masiteng PL (2022) Synthesis and investigation of energy transfer mechanisms in Sm^3+^ and Eu^3+^ doped Na_6_Mg(SO_4_)_4_ nanophosphors via solution combustion technology. Inorg Nano-Metal Chem 2081196

[26] Deyneko DV, Titkov VV, Fedyunin FD, Spassky DA, Volkov SN, Borovikova EY, Lazoryak BI, Aksenov SM (2022) Ellestadite-type anionic [PO_4_]^3-^→[SO_4_]^2-^ substitutions in β-Ca_3_(PO_4_)_2_ type compounds: A new route to design the inorganic phosphors. Ceram Int 48:24012–24020

[27] Petricek V, Dusek M, Palatinus L, Petricek V, Dusek M, Palatinus L (2014) Crystallographic computing systems JANA2006: general features. Zeitschrift Krist 229:345–352

[28] Bubnova RS, Firsova VA, Volkov SN, Filatov SK (2018) RietveltToTensor: Program for processing powder X-ray diffraction data under variable conditions. Glas Phys Chem 44:33–40

[29] Guo Q, Ma B, Liao L, Molokeev MS, Mei L, Liu H (2016) Crystal structure and luminescence properties of novel Sr_10-x_(SiO_4_)_3_(SO_4_)_3_O:xEu^2+^ phosphor with apatite structure. Ceramic International 42:11687–11691

